# Interactions and Conformational Plasticity of Human Uncoupling Protein 1 in Response to Small-Molecule Analogues: Insights from Molecular Dynamics Simulations

**DOI:** 10.1021/acsomega.6c01124

**Published:** 2026-06-11

**Authors:** Sanket Rathod, Utkarsh A. Jagtap, Falguni Pankhania, Ravi Shukla, Atish T. Paul

**Affiliations:** † Laboratory of Natural Product Chemistry, Department of Pharmacy, 29794 Birla Institute of Technology and Science, Pilani, Pilani Campus, Vidya Vihar, Pilani, Rajasthan 333031, India; ‡ School of Science, 5376 RMIT University, Melbourne, VIC 3000, Australia; § Biomedical Research, PK Sciences, Novartis Healthcare Private Limited, Hyderabad, Telangana 500032, India; ∥ NanoBiotechnology Research Laboratory, Centre for Advanced Materials & Industrial Chemistry, 5376 RMIT University, Melbourne, VIC 3000, Australia

## Abstract

Obesity is a multifactorial metabolic disorder associated with excess adiposity and risk of comorbidities. Uncoupling protein 1 (UCP1) is a mitochondrial inner-membrane carrier enriched in brown and beige adipose tissue. UCP1 dissipates the proton motive force to drive nonshivering thermogenesis, making it an attractive target for antiobesity drug discovery. However, the molecular determinants governing small-molecule engagement of UCP1 remain incompletely defined. Here, we applied an integrated *in silico* workflow to assess whether antiobesity pancreatic lipase inhibitory scaffolds can stably bind within the central cavity of UCP1 and modulate cavity-associated structural dynamics. From an in-house library (∼300 PL inhibitors), descriptor-based filtering prioritized 11 analogues for modeling. Atomistic molecular dynamics simulations (100 ns per system; extended to 1 μs for the top candidate) were then performed for UCP1 complexes and interpreted relative to benchmark ligand-bound states. Several analogues displayed persistent cavity occupancy and reproducible contacts with conserved residues, including the arginine network implicated in ligand recognition. Among these, BITSNPRG57 showed the most stable binding behavior over simulation time and was associated with relaxation of the conserved arginine triplet geometry. BITSNPRG57 also formed recurring water-mediated contacts with Asp27/28, a residue previously linked to fatty-acid-associated proton-handling motifs. Microsecond-scale simulations further supported the stability of the BITSNPRG57–UCP1 complex and the persistence of key interaction networks, including a sustained hydrogen bond to Ser229. Collectively, these results provide mechanistic structural hypotheses for small-molecule modulation of UCP1 and identify BITSNPRG57 as a promising scaffold for further optimization and experimental validation.

## Graphical abstract

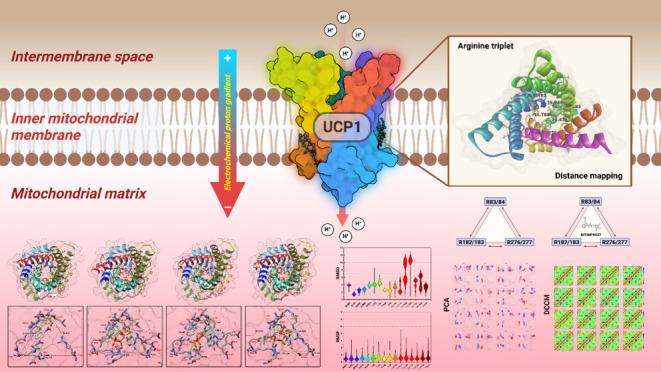

## 1 Introduction

Obesity is a multifactorial metabolic disorder driven by chronic energy imbalance and excess adiposity, and it remains a major global public health challenge.^1,2^ Overweight and obesity are commonly classified using body mass index (BMI), with BMI ≥ 25 kg/m^2^ indicating overweight and BMI ≥ 30 kg/m^2^ indicating obesity.^3,4^ Global prevalence has risen sharply since 1990 across age groups, as documented in large-scale analyses, including the NCD Risk Factor Collaboration report in The Lancet, which highlights an increasing worldwide burden dominated by rising obesity rates.^2,5,6^ This trend underscores the need for improved preventive and therapeutic strategies and for a mechanistic understanding of molecular pathways underlying energy intake, storage, and expenditure.

Dietary fat processing is a key contributor to positive energy balance (Figure 1). Dietary triglycerides constitute the majority of fat intake and are hydrolyzed predominantly by pancreatic lipase (PL), enabling the absorption of fatty acids (FA) and monoacylglycerols, followed by their re-esterification and storage in adipose tissue. Pharmacological inhibition of PL, therefore, represents an established strategy to reduce lipid absorption; for example, orlistat reduces dietary fat uptake by blocking PL-mediated hydrolysis.^7–10^ In parallel with reducing energy intake, increasing energy expenditure provides a complementary therapeutic axis. Nonshivering thermogenesis in brown and beige adipose tissue increases energy dissipation, and this process is critically dependent on uncoupling protein 1 (UCP1), a mitochondrial inner membrane carrier (SLC25 family) that dissipates the proton motive force as heat rather than coupling it to ATP synthesis.^11,12^

**Figure 1:**
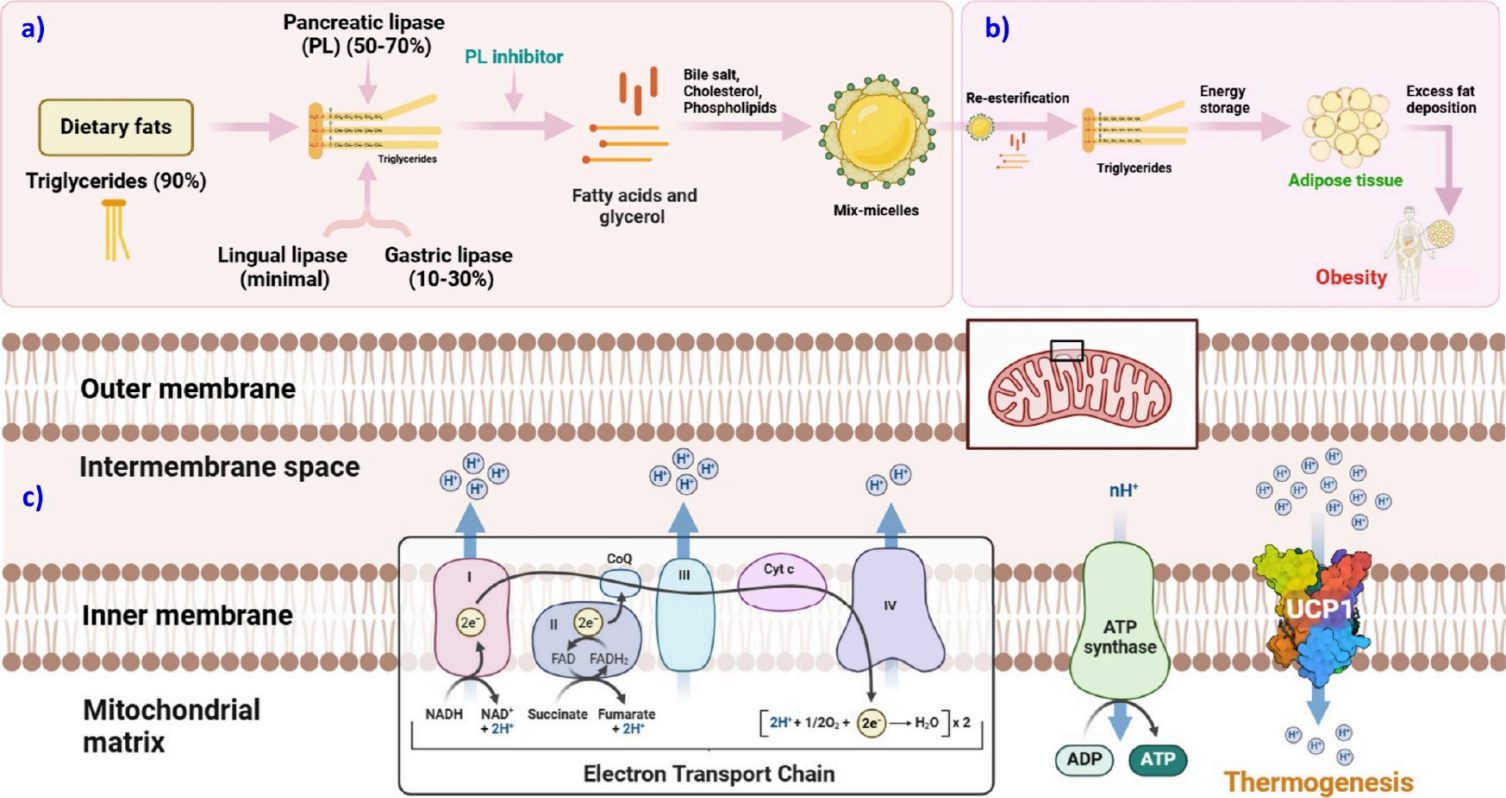
Schematic representation of energy (fat) intake, storage, expenditure, and mitochondrial thermogenesis mediated by UCP1. (a) Dietary triacylglycerols (major component of dietary fat) are hydrolyzed predominantly by PL following limited initial digestion by lingual and gastric lipases. PL inhibition reduces triacylglycerol hydrolysis and limits the generation of absorbable FAs and glycerol. (b) Absorbed FAs and monoacylglycerols are re-esterified to triacylglycerols in enterocytes, transported in chylomicrons, and stored in adipose tissue; sustained storage contributes to adipose expansion and obesity. (c) In brown adipose tissue mitochondria, the electron transport chain generates a proton motive force across the inner mitochondrial membrane; UCP1 dissipates this gradient as heat (proton leak) rather than coupling it to ATP synthesis, supporting nonshivering thermogenesis and increased energy expenditure. Created with BioRender.com.

Physiologically, UCP1 activity is stimulated by free FA generated during adrenergic-driven lipolysis, whereas purine and pyrimidine nucleotides are well-established endogenous inhibitors. Although synthetic protonophores such as 2,4-dinitrophenol (DNP) have historically been linked to thermogenesis, their lack of specificity and the extent of UCP1 dependence remain debated, limiting translational relevance.^13–15^ Moreover, despite the central role of FAs in UCP1 activation, high-resolution experimental structures capturing FA-bound UCP1 states remain unresolved, leaving key aspects of ligand recognition and gating incompletely defined at the molecular level.^16–19^

Beyond direct modulation of UCP1 activity, many interventions enhance thermogenesis indirectly by increasing UCP1 expression during browning of white adipocytes. A range of natural products and phytochemicals have been reported to upregulate UCP1 expression.^20^ Several also exhibit PL inhibitory activity, raising interest in dual-action antiobesity strategies that can influence both energy intake and expenditure. However, only a limited number of small molecules have been proposed to directly modulate or activate UCP1 at the protein level,^21–23^ highlighting a gap in the mechanistic understanding of the determinants of small-molecule binding and ligand-induced conformational effects in UCP1.

Recent availability of experimental UCP1 structures enables structure-based interrogation of ligand binding and conformational modulation. Building on our previous work establishing physicochemical and absorption-guided filters based on reported UCP1 expression-enhancing small-molecule analogues,^24^ here we investigated whether potent in-house PL inhibitory analogues can potentially engage the UCP1 central cavity and modulate its conformational dynamics. We curated an in-house library (∼300 PL inhibitory analogues) using potency (IC_50_) and a LogP/intestinal absorption filter, and then evaluated UCP1 in apo form, in DNP- and nucleotide-bound states, and in complexes with selected analogues using atomistic molecular dynamics (MD) simulations. Through comparative analysis of binding stability, residue-level interaction networks, cavity geometry, and protein dynamics, this study aims to define structural signatures associated with analogue binding. Furthermore, the study seeks to identify scaffolds warranting further experimental investigation as potential dual-target modulators of lipid absorption and thermogenic regulation.

## 2 Results and Discussion

The nucleotide-free (apo) state structure of human UCP1 (PDB: 8HBV), along with ligand-bound structures, DNP-bound (8J1N), ATP-bound (8HBW), GTP-bound (8G8W), and UTP-bound (9FZQ), were retrieved from the RCSB Protein Data Bank.^12,25,26^ These experimentally resolved states were used as benchmarks to contextualize ligand-binding modes and to guide the preparation of UCP1 complexes with selected in-house analogues for subsequent analysis of structural stability and conformational sampling. UCP1 belongs to the mitochondrial carrier (SLC25) family and is proposed to sample cytoplasmic-facing (c-state) and matrix-facing (m-state) conformations through coordinated rearrangements of transmembrane (TM) helices and conserved salt-bridge networks.^27,28^ Recent cryo-EM structures have expanded the structural basis for computationally interrogating UCP1 ligand recognition and inhibition/activation models. Purine nucleotides (e.g., ATP and GTP) are well-established inhibitors of UCP1, and recent structural work further indicates that pyrimidine nucleotides (e.g., UTP and dTTP) also bind UCP1 in a pH-dependent manner through phosphate-driven electrostatic interactions.^26^ Despite these advances, direct binding and protein-level modulation of UCP1 by many reported small-molecule “UCP1 activators” (often identified as transcriptional enhancers) remains insufficiently defined.^20,23,29–31^

The overall architecture of UCP1 and the positioning of native ligands across available structures are summarized in Figure 2a–c. A key structural feature in the central cavity is the conserved arginine network, widely implicated in anion recognition and gating in mitochondrial carriers.^32–34^ Residue numbering differs between PDB entries: in 8HBV/8J1*N*/8HBW the triplet is annotated as Arg83, Arg182, and Arg276, whereas in 8G8W/9FZQ (UniProt P25874 numbering) the equivalent residues are Arg84, Arg183, and Arg277. This mapping was applied consistently throughout all analyses. The location of this arginine network within the TM bundle is shown for the apo structure (Figure 2d). In available nucleotide-bound structures, these residues contribute to a strongly electropositive cavity region that stabilizes anionic ligands (e.g., nucleotide phosphates and fatty-acid anions) and is coupled to matrix-side gating behavior. Consequently, ligand-dependent changes in the spatial relationships among these residues provide a structural readout of cavity remodeling that may influence inhibitory or permissive conformational ensembles.^14,27,34^

**Figure 2:**
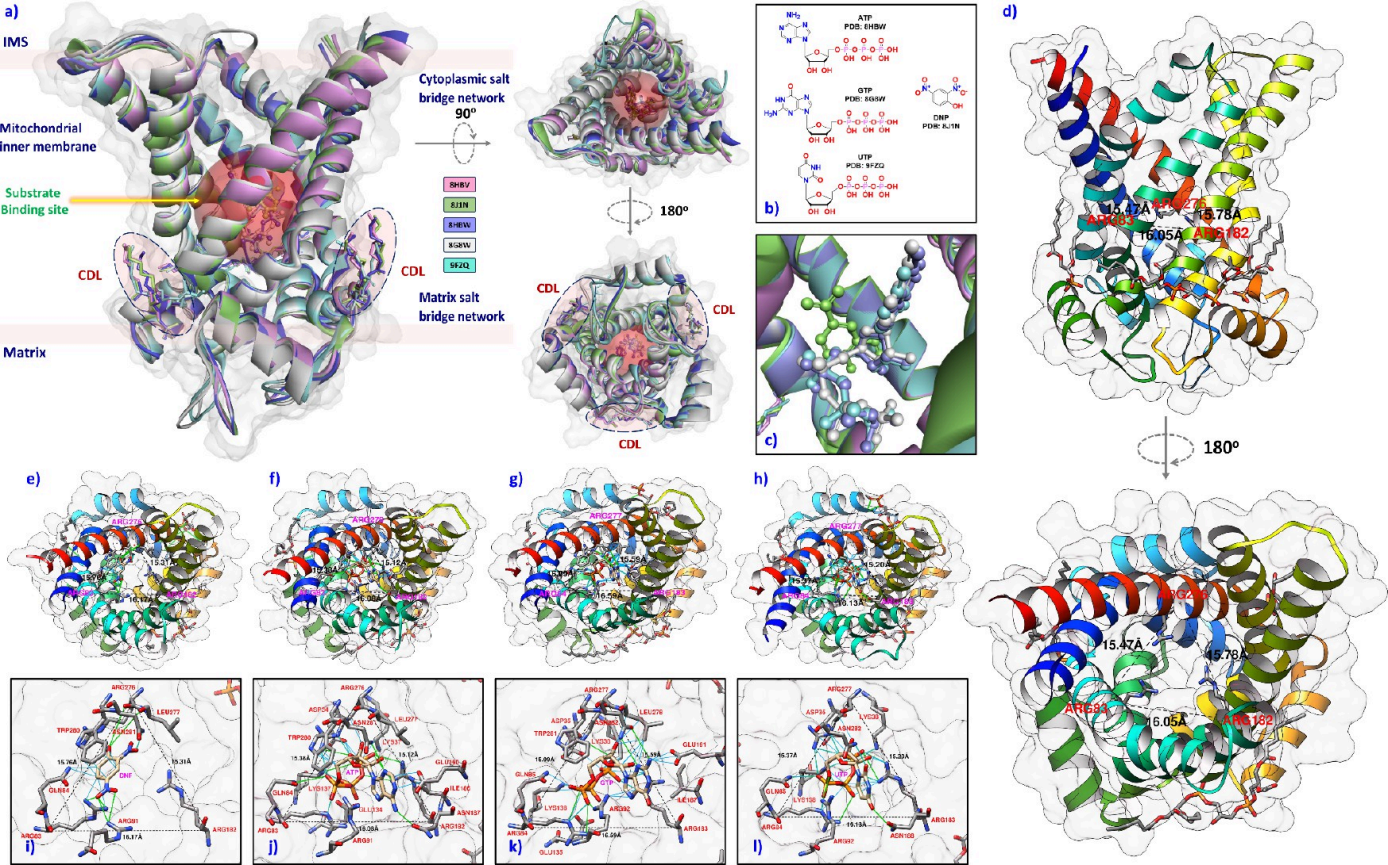
Structural benchmarks for UCP1 conformational states and native ligand binding. (a) Superposition of human UCP1 structures in the apo and ligand-bound states (apo, DNP-, ATP-, GTP-, and UTP-bound), highlighting the central cavity (red sphere) and resolved cardiolipin (CDL) binding sites (circled). Side and rotated (180°) views illustrate cytosolic-facing and matrix-facing orientations. (b) Chemical structures of benchmark (native) ligands corresponding to the reported complexes: ATP (8HBW), GTP (8G8W), UTP (9FZQ), and DNP (8J1N). (c) Overlay of native ligands within the substrate-binding cavity. (d) Side and cytosolic-facing views of UCP1 (8HBW) showing the conserved arginine network and the Cα–Cα distances measured between arginine residues. (e–h) Pairwise distances within the conserved arginine triplet for DNP-, ATP-, GTP-, and UTP-bound structures. (i–l) Interaction maps of UCP1 with each native ligands. Residue numbering in the deposited UCP1 structures varies among available PDB structures; notably, only PDB IDs 8G8W and 9FZQ align with the UniProt sequence (P25874). For consistency, all residue numbers used in this study correspond to those assigned in the original PDB files without adjustment.

Consistent with this, inter-residue distance measurements extracted from the native ligand-bound structures indicate ligand-dependent modulation of the arginine-network geometry (Figure 2e–h), and the corresponding interaction patterns for DNP and nucleotides are shown in Figure 2i–l. These benchmark states were therefore used as reference points for interpreting the conformational and interaction signatures observed in the in-house analogue–UCP1 complexes. By comparing analogue-bound systems with apo and nucleotide-bound ensembles, we sought to identify ligand-dependent structural patterns associated with stable cavity engagement and altered central-cavity geometry.

### 2.1 Ligand Selection

A systematic, data-driven curation of small-molecule candidates was performed from an in-house library of PL inhibitors to prioritize analogues for UCP1 binding and dynamics studies. Small-molecule analogue management and filtering were carried out in DataWarrior. An initial set of 320 PL inhibitory analogues with experimentally reported PL inhibitory potency (IC_50_) was screened, and a potency threshold of IC_50_ < 5 μM was applied to focus on the most active candidates, yielding 27 analogues. To further prioritize analogues with physicochemical properties compatible with oral exposure and membrane access, the 27 candidates were filtered using a LogP/intestinal absorption criterion adapted from our recent work.^24^ LogP and intestinal absorption were predicted using ADMET Predictor (v10.0.0.0; GastroPlus, Simulations Plus, USA). Analogues were retained if they satisfied LogP = 3–6 and had predicted intestinal absorption >90%, yielding a final set of 11 analogues. The chemical structures of the selected compounds are shown in Figure 3.

**Figure 3:**
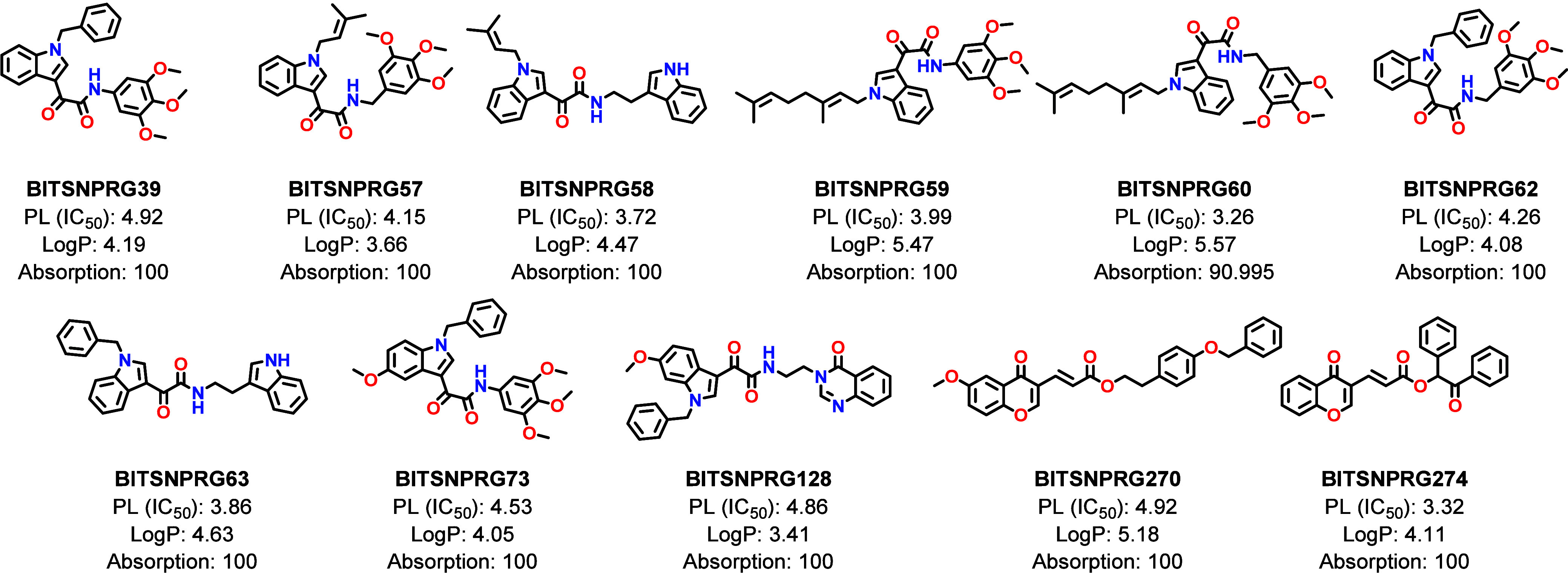
Chemical structures of selected PL inhibitory analogues for the current investigation. The structures are shown along with their respective IC_50_ values (μM) for PL inhibition, predicted lipophilicity (LogP predictions via ADMET Predictor v10.0.0.0, GastroPlus software, Simulations Plus, Lancaster, CA, USA), and intestinal absorption (QikProp, Schrödinger, LLC, New York, NY, 2025).

For subsequent modeling, each of the 11 analogues was geometry-optimized using density functional theory (DFT) in ORCA to obtain low-energy conformations for docking (protein–ligand complex preparation) and to support electronic-structure analysis. In addition to gas-phase calculations, geometry optimizations were performed in an implicit-solvent environment. The resulting gas- and solvent-phase geometries were highly similar, as quantified by RMSD analysis; superimposed structures for all analogues are provided in the Supporting Information (Figures S3–S13). All the optimizations were performed using the B3LYP/def2-TZVP level of theory with RIJCOSX acceleration and D4 dispersion correction (OPT RIJCOSX D4).^35,36^ Output structures were inspected using the ORCA-enabled Avogadro interface. Additional computational details are provided in the Supporting Information.

### 2.2 Assessment of Prepared Complexes

Docking was used solely to generate initial binding poses for selected in-house analogues within the central cavity of UCP1 prior to MD simulations (Figures S14–S24). Pose generation and selection were guided by experimentally informed ligand-binding features reported for UCP1, with emphasis on the matrix-side cavity region implicated in nucleotide and small-molecule binding. Complex preparation was based on the DNP-bound cryo-EM structure of UCP1 (PDB: 8J1N), which provides a resolved reference for small-molecule occupancy within the central cavity. Nonprotein components (e.g., nanobodies/sybodies) were removed to minimize external structural constraints and to focus the simulations on intrinsic UCP1–ligand interactions. Residues lining the cavity and repeatedly implicated in ligand recognition in 8J1N and related structures, including Arg83/Gln84, Arg91, Arg182, Ile186, Arg276/Leu277, and Trp280, were used to define and evaluate the docking region. Using the DNP pose as a spatial reference, each analogue was docked into the cavity to generate multiple candidate orientations (10 poses per ligand). The resulting poses were screened by (i) inspection of cavity-appropriate interaction patterns (electrostatic and polar contacts with conserved residues, and hydrophobic/π interactions with TM6-adjacent residues) and (ii) MM-GBSA rescoring to prioritize energetically favorable binding modes. For each ligand, the pose exhibiting the most plausible interaction fingerprint together with the desired MM-GBSA binding energy profile was selected as the starting configuration for MD. In the selected starting complexes, all analogues occupied the central carrier cavity and overlapped with the binding region observed for native ligands in available UCP1 structures. The prepared complexes reproduced stabilizing noncovalent contacts (salt bridges/polar interactions and hydrophobic/π contacts) with key cavity residues, supporting their suitability for subsequent dynamic analysis. Representative 3D interaction diagrams and energy profiles for the final selected poses are provided in Figure S1.

### 2.3 Molecular Dynamics Simulation

Atomistic MD simulations were performed using Desmond software to characterize the structural dynamics of UCP1 in multiple ligand-bound states. Although UCP1 is central to mitochondrial thermogenesis, the molecular basis of its conformational plasticity and gating remains incompletely defined. Recent work by Vojvodić et al. has clarified how UCP1 facilitates fatty acid transport across the inner mitochondrial membrane, providing strong support for the fatty acid cycling model first proposed by Skulachev. Using a combination of simulations and functional experiments, they showed that fatty acid anions travel along specific paths at the protein–lipid interface to reach key arginine residues in UCP1.^37^ These residues are crucial for transport and are also conserved in other SLC25 proteins, suggesting that a similar fatty acid transport mechanism may operate more widely across this carrier family. In another study, Jacobsen et al. proposed a model in which a protonated fatty acid traverses a central binding site, coordinated by Asp28 and water molecules, and further showed that nucleotide binding perturbs this process through TM helix rearrangements, as supported by simulation and mutagenesis.^38^ Building on this framework, we used atomistic MD simulation to interrogate how a panel of in-house PL inhibitory analogues modulate the UCP1 central cavity relative to benchmark ligand states. UCP1 complexes were prepared for (i) in-house analogues and (ii) native ligands, including DNP and endogenous nucleotides, to enable direct structural comparison across ligand classes. Each system was simulated for 100 ns, and trajectories were saved at uniform intervals (2000 frames per trajectory) to support time-resolved analyses. Postprocessing and quantitative analysis were then performed using standard structural metrics (e.g., RMSD/RMSF, radius of gyration, inter-residue distances, and interaction fingerprints) to evaluate global stability, local flexibility, and ligand-associated modulation of cavity geometry. While these 100 ns simulations provide an initial screening of pose stability and ligand effects, a more complete characterization of UCP1 conformational plasticity will require longer-time scale MD simulations.

#### 2.3.1 Conformational Pattern and Compactness

Conformational stability of UCP1 in the presence of native ligands and in-house analogues was assessed using RMSD of the protein Cα atoms and ligand RMSD calculated after alignment of each trajectory to the UCP1 backbone. Protein Cα RMSD provides a global measure of structural deviation over time (Figure S25), whereas ligand RMSD (protein-aligned) reports the stability of the bound pose within the cavity (Figure S25). Comparative visualization of protein and ligand RMSD distributions (Figure 4) was used to distinguish ligand-associated conformational sampling from ligand mobility within (or away from) the binding pocket. Across all simulated complexes, UCP1 maintained Cα RMSD values below ∼5.5 Å over 100 ns, indicating preservation of the overall fold and absence of large-scale structural disruption under the simulated conditions (Figure S25). In contrast, ligand RMSD profiles were ligand-dependent, indicating variable pose stability and cavity retention across the compound set (Figures S25 and 4). Among the benchmark systems, DNP- and ATP-bound complexes exhibited comparatively narrow and stable RMSD distributions, consistent with persistent binding and limited conformational drift. The GTP-bound system (8G8W) displayed a broader RMSD distribution for the protein, indicating greater conformational heterogeneity within the simulation window. The UTP-bound complex (9FZQ) showed a more constrained protein RMSD profile, with stabilization around ∼4 Å, although the distribution suggests sampling of multiple conformational substates (Figure 4).

**Figure 4:**
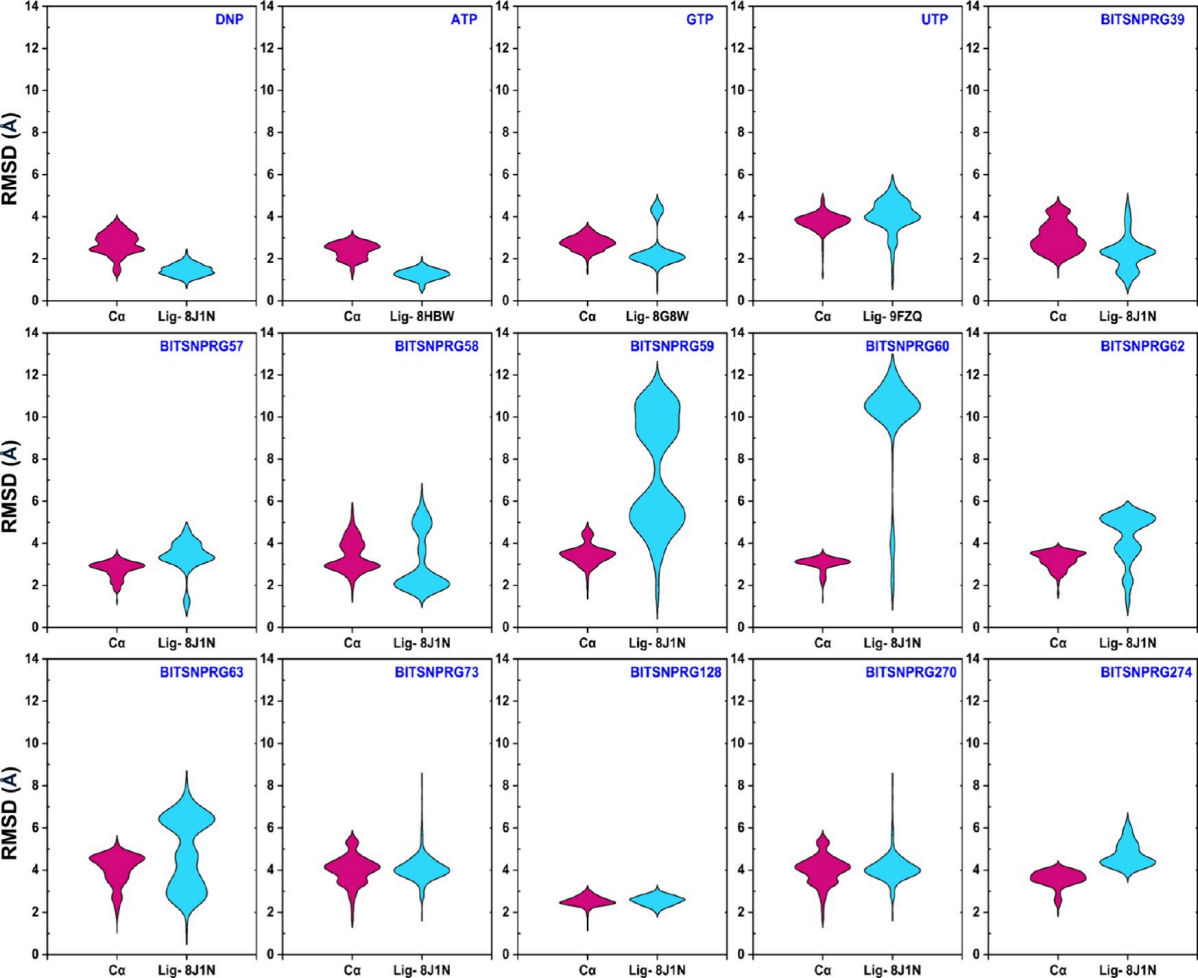
Conformational pattern over 100 ns studied with RMSD. Violin plots summarizing comparative analysis of RMSD distributions between Cα atoms (magenta violin) and ligand-bound pocket (cyan violin) across the trajectory for each complex.

For the analogue-bound systems, most complexes remained within a protein Cα RMSD range of ∼2–6 Å, again supporting global stability of the UCP1 fold. However, ligand RMSD distributions separate analogues with persistent cavity engagement from those exhibiting substantial mobility. BITSNPRG57, BITSNPRG73, and BITSNPRG128 showed comparatively compact ligand RMSD distributions (typically ∼2–4 Å), consistent with stable pose retention and sustained cavity occupancy. These profiles were like the DNP-bound benchmark, suggesting good geometric compatibility with the central cavity environment. BITSNPRG39, BITSNPRG270, and BITSNPRG274 showed intermediate behavior, with overall pocket retention but occasional excursions; BITSNPRG270 displayed transient increases in protein Cα RMSD (up to ∼6 Å) without evidence of global destabilization. In contrast, BITSNPRG58, BITSNPRG59, BITSNPRG60, BITSNPRG62, and BITSNPRG63 exhibited broader and/or multimodal ligand RMSD distributions (often >10 Å), consistent with reduced pose stability and increased ligand mobility relative to the protein scaffold. Notably, BITSNPRG59 and BITSNPRG60 showed large ligand RMSD despite stable protein Cα RMSD (<5 Å), supporting ligand displacement and/or reorientation within the cavity rather than protein-driven structural drift. Collectively, the RMSD analysis identifies BITSNPRG57, BITSNPRG73, and BITSNPRG128 (followed by BITSNPRG39, BITSNPRG270, and BITSNPRG274) as the most stable binders within this analogue set over the 100 ns simulations, motivating deeper interaction and long-time scale analyses for these candidates.

#### 2.3.2 Structural Flexibility and Residual Fluctuations

RMSF analysis was performed across the simulation trajectories to evaluate the local flexibility and per-residue dynamics of UCP1 Cα in both the ligand-bound and ligand-free states (Figure 5). Overall, all ligand-bound complexes, including native and PL-inhibitory analogues, displayed a comparable fluctuation pattern. The pronounced peaks in RMSF are observed near the N- and C-terminal regions, which are typically more solvent-exposed and structurally flexible. Notably, residues in the central TM helices exhibited minimal fluctuations. This pattern reflects the structural rigidity essential for the functional core of UCP1. The spiked pattern in the violin also appeared due to the fluctuation shown by the amino acids of the central TM. Complexes with ligands such as BITSNPRG57 and BITSNPRG60 showed reduced fluctuations in the TM domains. However, the BITSNPRG60-bound complex exerted an observable structural change.

**Figure 5:**
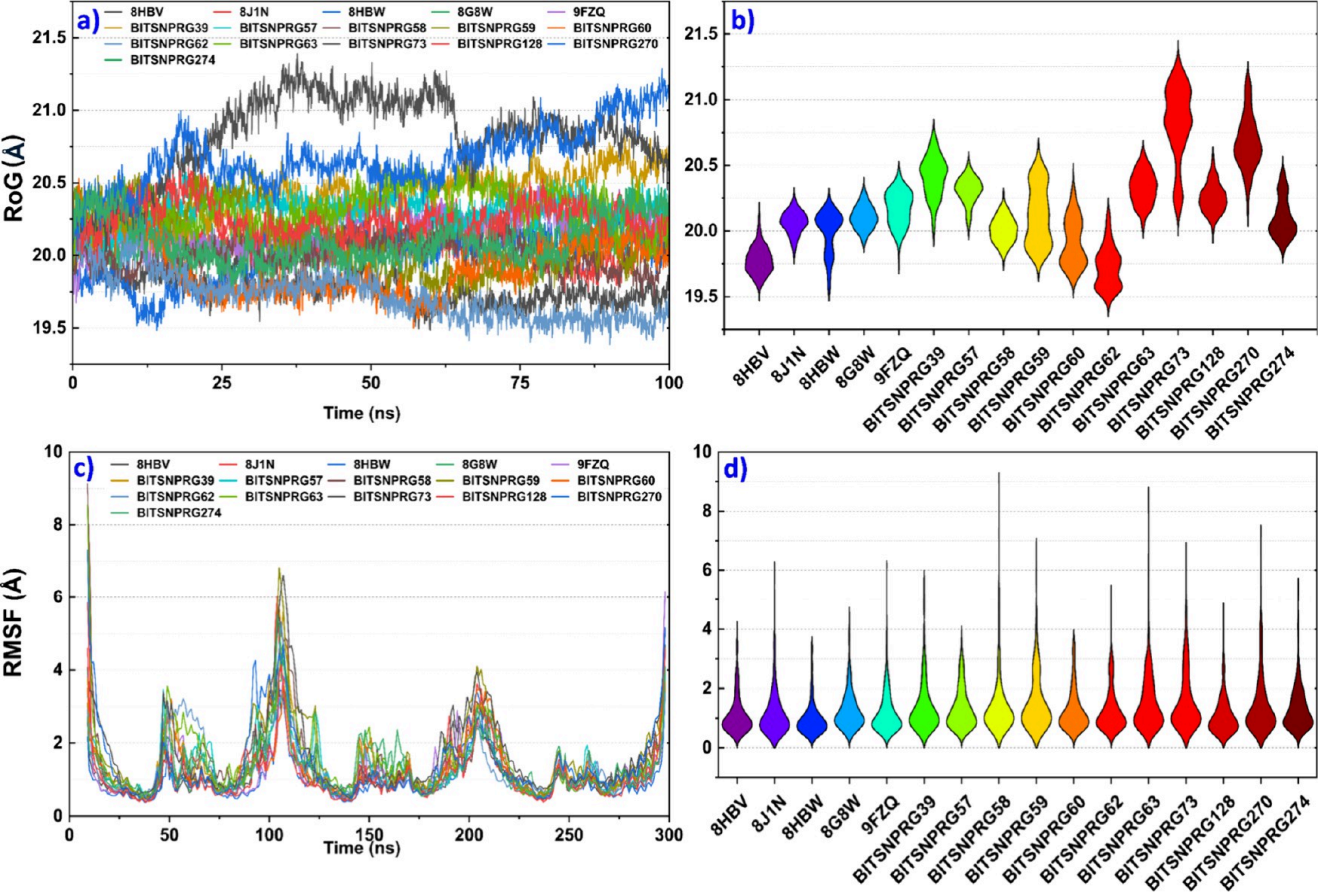
Global structural compactness and local flexibility of UCP1 in ligand-free and ligand-bound states. (a) RoG profiles showing the time-resolved evolution of structural compactness for UCP1 Cα atoms across all simulated complexes. (b) Violin plots of RoG distributions depicting the global conformational spread and stability of the protein in each ligand-bound or reference state. (c) RMSF plots highlighting per-residue flexibility across UCP1, distinguishing mobile loop regions from structurally rigid TM helices. (d) Corresponding violin plots of RMSF values capturing the variance in local residue dynamics across simulation trajectories.

The BITSNPRG57-bound complex exhibited a stable RMSD profile across the simulation. It maintained consistent structural integrity throughout, without significant deviations. The RoG analysis further supported this stability by indicating a relaxed yet compact conformation (Figure 5). Compared to the native state, this conformational behavior suggests that BITSNPRG57 may stabilize UCP1 in a potentially functional state. Ligands such as BITSNPRG58, BITSNPRG59, BITSNPRG60, BITSNPRG63, and BITSNPRG73 showed slightly elevated fluctuations. For some of them, the RMSF profile aligns with their higher RMSD and RoG values, indicating possible local destabilization. These observations were further verified by the RMSF violin plots, where narrower and lower-distribution violins were evident for residual stability in the complexes. Together, the RMSF profiles support the notion that certain PL-inhibitory analogues impose structural restraint on key domains of UCP1, which may influence its conformational dynamics and regulatory behavior.

#### 2.3.3 Dynamics of Arginine Triplet

To quantify ligand-dependent modulation of the conserved arginine gating network, we monitored the time evolution of pairwise distances within the arginine triplet across the MD trajectories. Distances were computed for the three arginine–arginine pairs forming the central cavity network, Arg83/84–Arg182/183, Arg182/183–Arg276/277, and Arg276/277–Arg83/84 (across the compared structures), using distance metrics sampled over the 100 ns simulations. Distributions are summarized as violin plots in Figure 6, where the width reflects the probability density of distances populated during the trajectory and therefore reports both the dynamic range and the dominant conformational basin(s) of the triplet.

**Figure 6:**
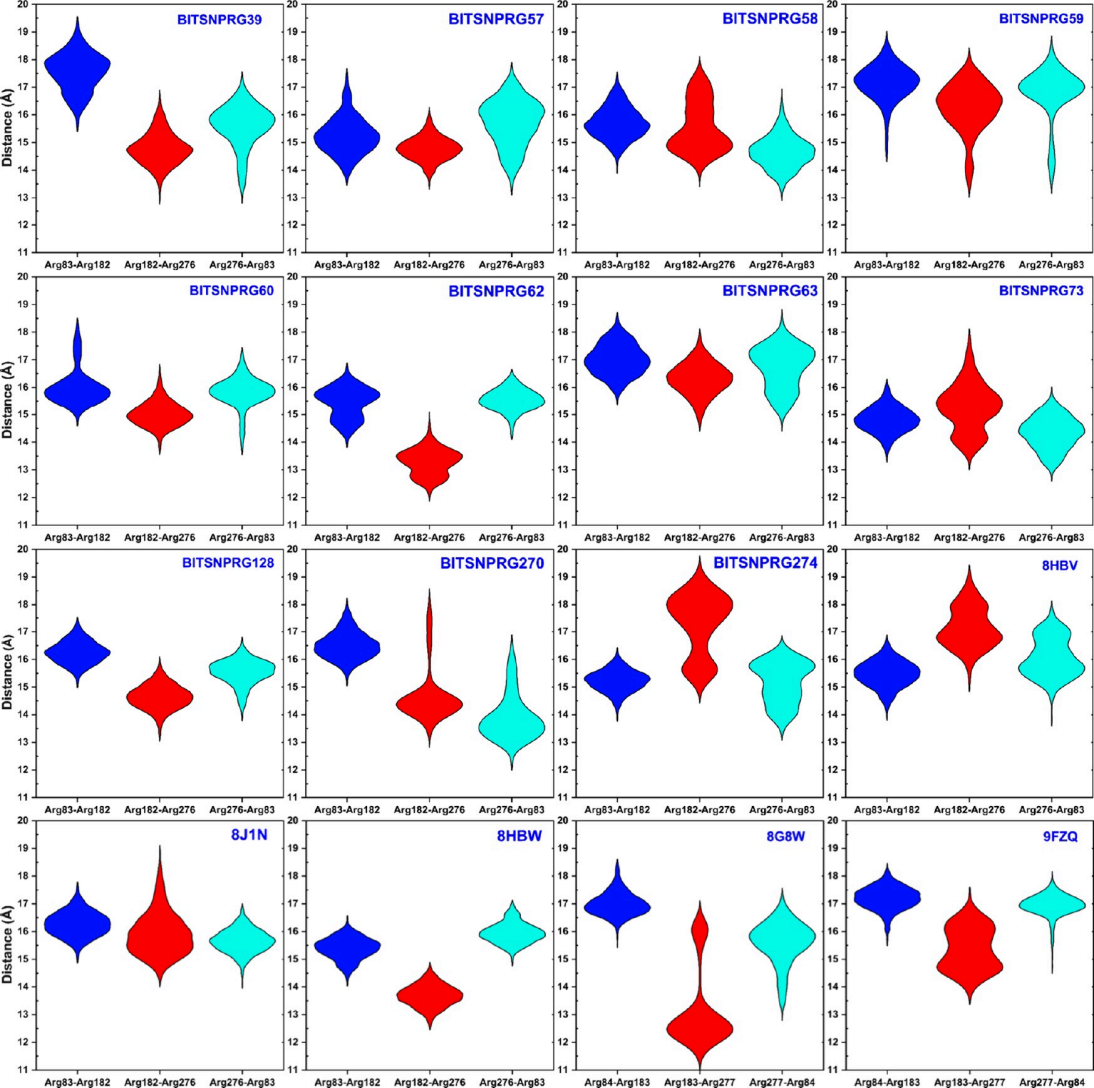
Distribution of inter-residue distances among arginine triplets in UCP1 across multiple complexes. Violin plots depict the distribution of pairwise distances (in Å) among key arginine residues forming the conserved triplet (Arg83/84, Arg182/183, Arg276/277) in UCP1 and its homologues, as obtained from MD simulations of distinct ligand-bound complexes. Each subplot corresponds to an individual complex, showing the dynamic behavior of the residue pair distances over the simulation trajectory. The width of each violin reflects the relative frequency of observed distances. Color coding denotes specific residue pairs within the triplet: blue for Arg83/84–Arg182/183, red for Arg182/183–Arg276/277, and cyan for Arg276/277–Arg83/84, facilitating visual comparison across complexes.

In the nucleotide-free state (8HBV), the arginine network adopted a comparatively relaxed geometry, with all three pairwise distances predominantly populating the ∼14–20 Å range. Ligand binding shifted these distributions in a ligand-dependent manner. Among the benchmark ligands, the nucleotide-bound systems exhibited the most pronounced compaction. GTP-bound UCP1 (8G8W) showed the strongest contraction of the Arg183–Arg277 pair (frequently ≤ 12 Å), consistent with a more tightly packed cavity geometry relative to the apo ensemble. ATP (8HBW) and UTP (9FZQ) produced intermediate contraction, with UTP showing a stronger reduction in the Arg183–Arg277 and Arg277–Arg84 distances than ATP. In contrast, DNP-bound UCP1 (8J1N) maintained comparatively uniform distance distributions with limited asymmetric contraction, indicating that DNP does not stabilize the most compact arginine-network configuration observed for nucleotides over this time scale.

Within the analogue series, distinct “compacting” versus “relaxing” profiles were evident. BITSNPRG270 produced the most pronounced narrowing of the Arg182–Arg276 and Arg276–Arg83 distributions, indicating a persistent shift toward a more compact triplet geometry. BITSNPRG128, BITSNPRG39, BITSNPRG62, and BITSNPRG73 also displayed substantial contraction relative to the apo reference, in some cases approaching the compaction observed for the GTP-bound benchmark. BITSNPRG62 preferentially contracted the Arg182–Arg276 distance, consistent with a concerted tightening of one edge of the triplet. BITSNPRG128 showed a more asymmetric response (most evident for Arg276–Arg83), suggesting ligand-specific, nonuniform modulation of the triplet rather than a globally rigid “closure”. By contrast, BITSNPRG57, BITSNPRG58, BITSNPRG59, and BITSNPRG60 retained broader distance distributions closer to the apo-like ensemble, indicating a comparatively relaxed arginine-network geometry.

These distance-based fingerprints identify analogue-dependent shifts in the geometry and flexibility of the conserved arginine network, providing a structural readout of cavity remodelling induced by ligand binding. Given that the arginine triplet is coupled to central-cavity electrostatics and matrix-side gating, these patterns are consistent with ligand-specific biasing of UCP1 toward more compact or more open-like conformational ensembles under the simulated conditions. Interpretation of functional consequences should be considered alongside complementary interaction and hydration analyses, including Asp27/28-associated water-mediated contacts (see Section 2.3.5).

#### 2.3.4 Dynamic Correlation and Motion Analysis

Dynamic cross-correlation matrix (DCCM) analysis was used to assess how ligand binding alters coordinated motions within UCP1 during the MD simulations. DCCMs were computed from Cα-based residue–residue correlation behavior for 11 analogue-bound systems and five benchmark structures (PDB: 8HBV, 8J1N, 8HBW, 8G8W, 9FZQ). The resulting heatmaps (Figures S26–S37) report the extent to which residue pairs undergo concerted (positively correlated) or anticorrelated motion over the trajectory, thereby providing a residue-level view of dynamic coupling across the TM bundle.

Across all systems, the main diagonal remained sharply defined, and near-diagonal regions retained strong local correlations, consistent with the preservation of secondary structure and the absence of large-scale unfolding within the 100 ns time scale. Differences between ligands were instead reflected in off-diagonal correlation features and in the organization/density of correlated blocks spanning TM helices. Several analogues (BITSNPRG270, BITSNPRG274, BITSNPRG128, BITSNPRG39, BITSNPRG62, and BITSNPRG73) exhibited DCCM patterns indicative of increased compaction in the central TM region, consistent with reduced separation among residues constituting the conserved arginine network (e.g., Arg182–Arg276 and Arg276–Arg83). BITSNPRG128 additionally showed a more localized, asymmetric signature centered around residues ∼180–220, suggesting region-specific dynamic restriction rather than uniform global compaction.

In contrast, BITSNPRG57, BITSNPRG58, and BITSNPRG60 displayed more diffuse correlation patterns with reduced block-like compaction, consistent with comparatively higher internal mobility and broader sampling of cavity-adjacent conformations. Notably, complexes bound to BITSNPRG57 and BITSNPRG60 showed a distinct anticorrelation feature (cyan) within approximately residues 159–209, suggesting altered long-range coupling between this segment and the surrounding TM architecture. Compared with the benchmarks, nucleotide-bound structures (8HBW, 8G8W, 9FZQ) exhibited more compact, organized correlation blocks, whereas the apo (8HBV) and DNP-bound (8J1N) states showed greater flexibility and less constrained long-range coupling. Overall, the DCCM analysis complements the RMSD/RMSF/RoG metrics by describing how structural fluctuations are coordinated across the protein, rather than reporting only their magnitude.

To further characterize collective motions, principal component analysis (PCA) was performed on Cα coordinates for each trajectory. The first three principal components (PC1–PC3) captured the dominant variance in conformational sampling, and projections are shown in Figure S38. BITSNPRG58 and BITSNPRG59 exhibited the largest variance along PC1 (49% each), indicating that their trajectories were dominated by a single major collective motion. By contrast, BITSNPRG73 showed higher variance along PC2 (31.5%), consistent with a more distributed motion profile across multiple collective modes. Several analogues (e.g., BITSNPRG270 and BITSNPRG39) showed moderate contributions across PC1 and PC2, consistent with intermediate-amplitude collective rearrangements. In combination, the DCCM and PCA results demonstrate that the analogue set produces ligand-dependent changes in long-range coupling and collective motions of UCP1, consistent with distinct dynamical ensembles sampled within the simulation window.

#### 2.3.5 Interaction Analysis

Intermolecular interactions were quantified from the MD trajectories using residue–ligand contact maps, hydrogen-bond (H-bond) time series, and representative structural snapshots to assess how ligand binding reshapes the UCP1 central cavity. Across the 100 ns simulations, all in-house analogue–UCP1 complexes retained the ligands within the central carrier cavity and remained close to their starting poses from docking-based complex preparation, with conserved contacts maintained over time (Figure 7). To contextualize these interaction patterns, ligand-bound UCP1 crystal structures were used as structural benchmarks: DNP (PDB: 8J1N), ATP (8HBW), GTP (8G8W), and UTP (9FZQ).^12,25,26^ In these reference structures, residues including Lys38, Arg84, Arg91, Arg183, Lys138, and Arg277 recurrently contribute to stabilization within the cavity and matrix-gate region through electrostatic and polar interactions. The in-house analogue complexes reproduced several of these interaction hotspots, most consistently involving the conserved arginine triplet and Lys138, with additional polar contacts frequently observed for Glu191 and Asn282. Collectively, these data indicate that the analogues engage the core electrostatic network implicated in ligand recognition in UCP1, while also exhibiting ligand-specific contact topologies relative to DNP. The latter is consistent with differences in ligand size and geometry compared with DNP, which is smaller and therefore less likely to bridge multiple transmembrane (TM) segments. A notable interaction was observed for Asp28 (corresponding to Asp27 in 8J1N), a residue recently linked to water-mediated proton transfer in UCP1.^38^

**Figure 7:**
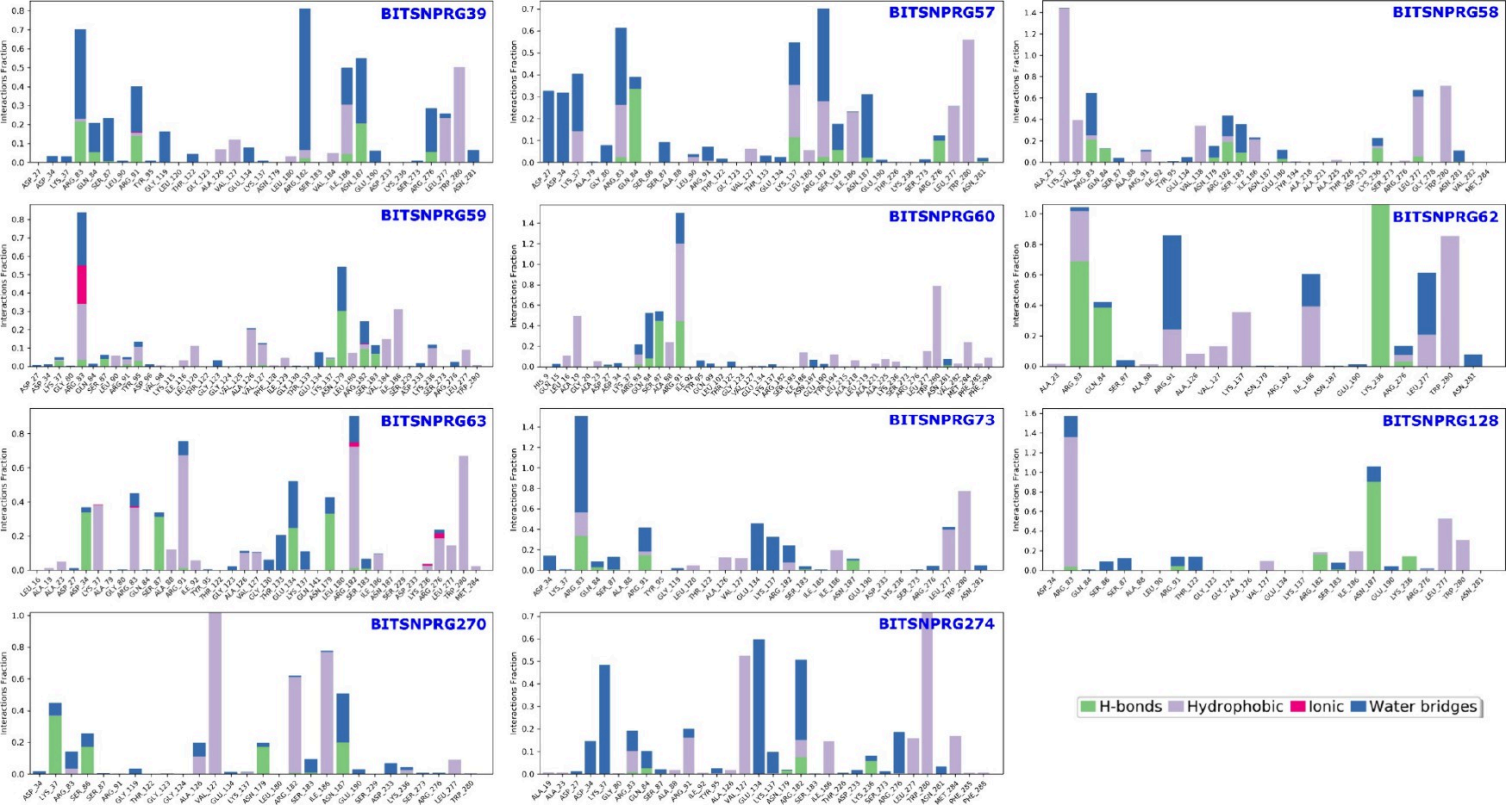
Categorization and frequency of protein–ligand interactions across simulation trajectories. Stacked bar charts depict the normalized occurrence of key noncovalent interactions between UCP1 residues and each ligand (PL inhibitory analogues) over the course of the simulation. Interaction types are classified into hydrogen bonds (green), hydrophobic contacts (purple), ionic interactions (magenta), and water bridges (blue), with each bar representing the cumulative contact frequency at a given residue. Normalization is performed according to default settings in Desmond and reflects the proportion of simulation time during which the interaction is maintained, with values greater than 1.0 indicating multiple concurrent contacts of the same type. Hydrogen bonds include backbone and side-chain interactions based on geometric criteria involving donor–acceptor distances (≤2.5 Å) and angular constraints (≥120° and ≥90°). Hydrophobic contacts encompass π–cation, π–π, and nonspecific aliphatic interactions within 3.6–4.5 Å. Ionic interactions involve oppositely charged atoms within 3.7 Å, excluding hydrogen bonds, while water bridges are characterized by a relaxed hydrogen-bond geometry mediated by solvent molecules.

In the BITSNPRG57-bound simulation, a persistent water-bridged interaction between the ligand and Asp28 was detected (Figure 8 and Video S1), whereas this feature was absent or substantially less frequent in the other analogue-bound systems. This identifies BITSNPRG57 as the analogue most closely reproducing the Asp28-centered hydration/contact motif proposed to be relevant for UCP1 proton-handling chemistry, within the limitations of classical MD. Hydrogen bonding was further quantified across all trajectories (Figure S39). The nucleotide-bound benchmark systems exhibited higher and broader H-bond distributions over time, with GTP showing the highest sustained H-bond counts, consistent with the extensive electrostatic complementarity of nucleotides to the cavity. DNP displayed markedly fewer H-bonds than nucleotides. The analogue-bound complexes generally exhibited intermediate H-bond counts, with distributions comparable to or modestly above those of DNP. Together, these analyses indicate that the in-house analogues maintain stable cavity occupancy and engage conserved UCP1 interaction hotspots, while BITSNPRG57 uniquely supports an Asp28-associated water-bridged contact pattern that may be relevant to conformational modulation.

**Figure 8:**
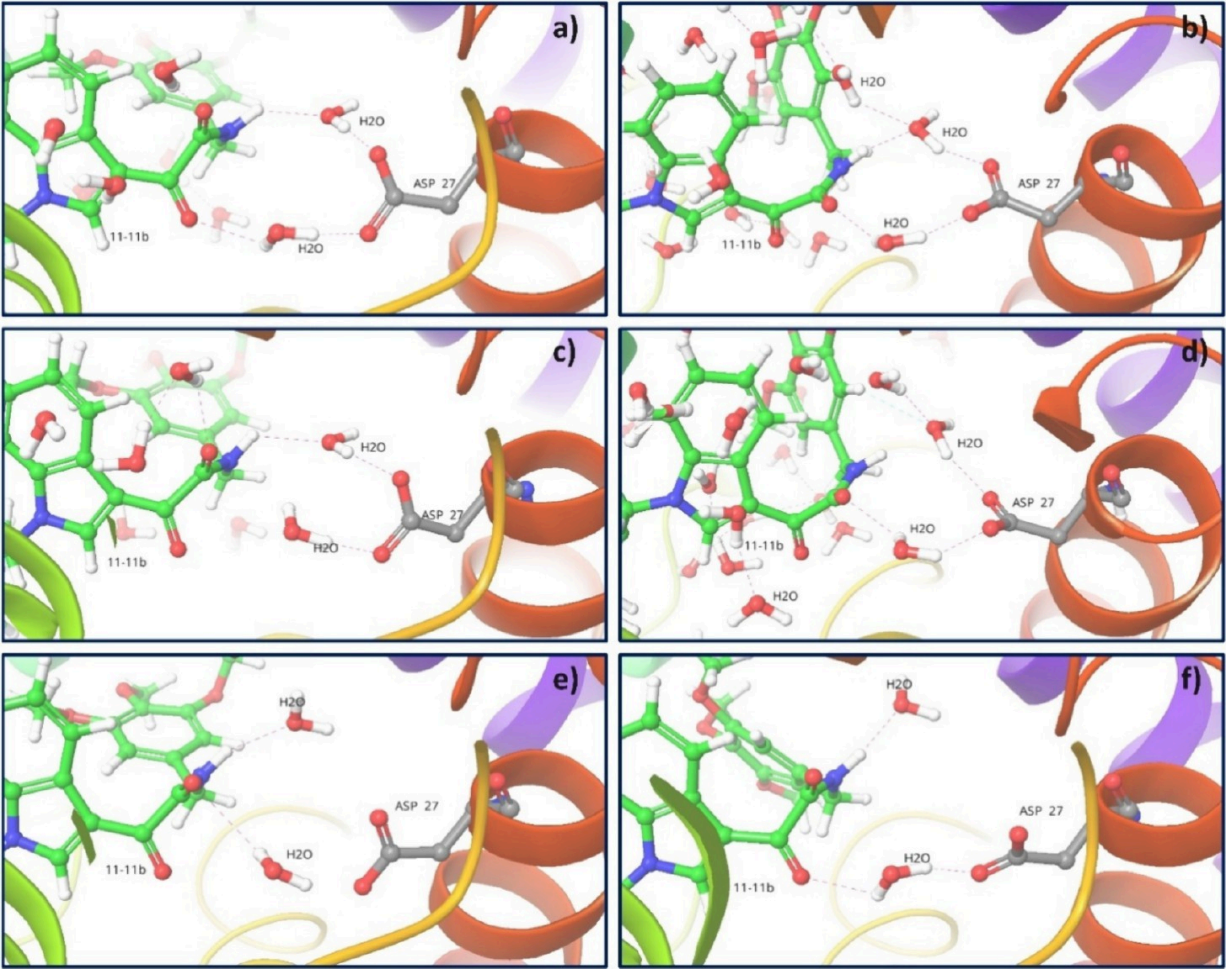
Water-mediated coordination of BITSNPRG57 with Asp28 in UCP1. Representative MD snapshot (a–f) showing the BITSNPRG57 ligand stably coordinated to Asp28 (Asp27 in PDB: 8J1N) via bridging water molecules within the central cavity of UCP1. These frames collectively suggest that BITSNPRG57 may structurally mimic key contacts, implicating Asp28 as a conserved mechanistic hotspot. Trajectory snaps captured in Schrödinger Maestro.

### 2.4 Potential of BITSNPRG57

BITSNPRG57 (chemical structure shown in Figure 9a) showed the most distinctive interaction fingerprint among the tested analogues in MD simulations of UCP1. Over 100 ns, the ligand remained stably bound in the central cavity and maintained recurrent contacts with conserved cavity residues. BITSNPRG57 exhibited a recurring water-mediated interaction with Asp27/28 (Asp27 in PDB: 8J1N numbering), a residue previously associated with fatty-acid-linked proton-handling motifs in UCP1.^14,27,38^ This Asp27/28-centered hydrated contact pattern was more persistent in BITSNPRG57 than in the other analogues-bound systems. In parallel, BITSNPRG57 was associated with a relaxation of the cavity geometry, reflected by increased separation among residues of the conserved arginine network (e.g., Arg83/84–Arg276/277) and reduced fluctuations in cavity-proximal metrics relative to several comparators. Benchmark comparisons to ATP-, GTP-, UTP-, and DNP-bound UCP1 structures indicated that BITSNPRG57 preserves key polar/electrostatic contacts observed in native ligand-bound states while introducing ligand-specific contacts consistent with its larger size and geometry. DFT analysis further indicated LUMO localization around the α-ketoamide region proximal to the indole motif (Figure S2), supporting its potential contribution to polar recognition within the cavity.

**Figure 9:**
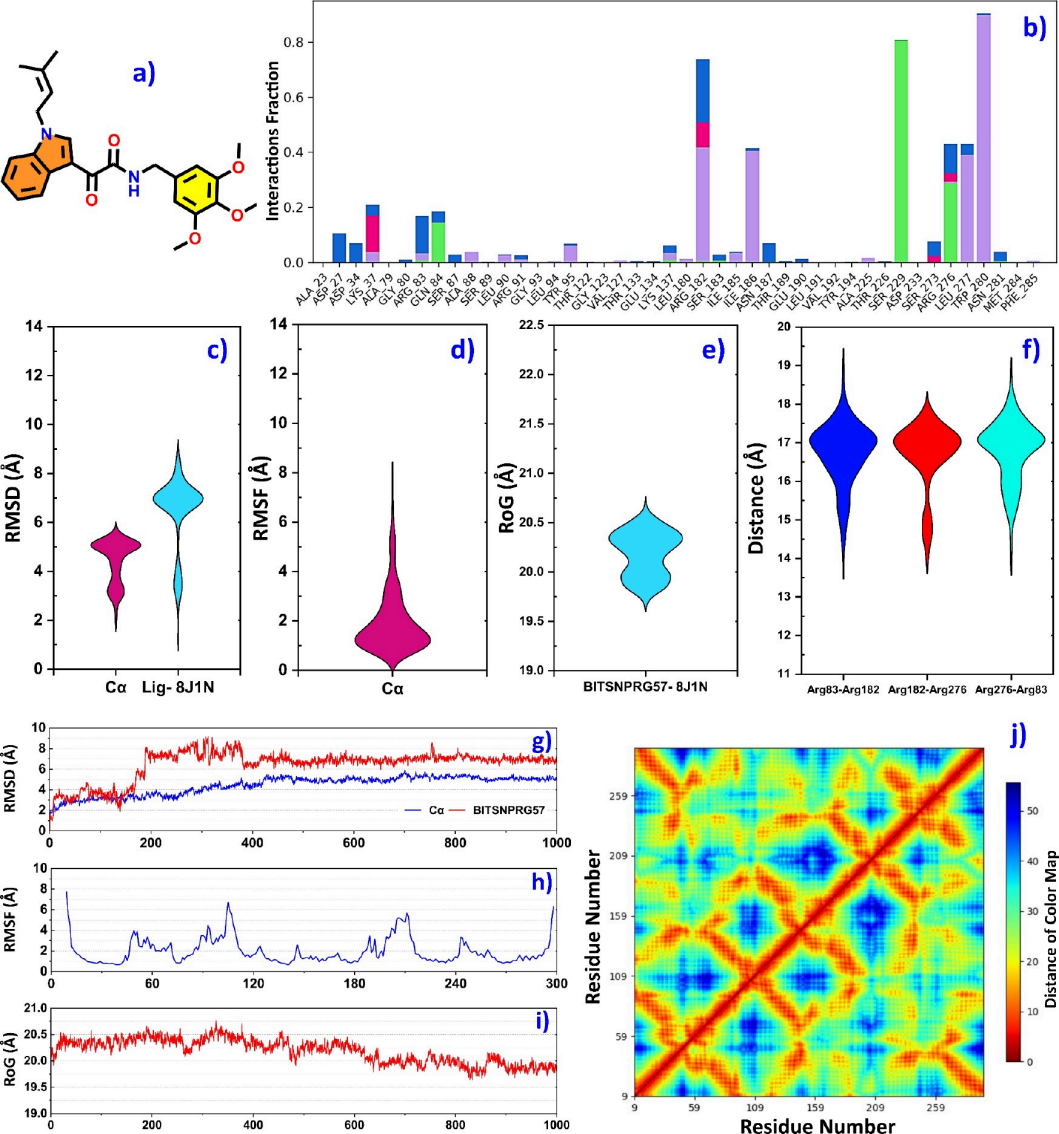
Statistical analysis of the extended simulation of BITSNPRG57-bound UCP1 complex over a 1 μs. (a) Chemical structure of BITSNPRG57. (b) Interaction fingerprint showing fraction and type of residue-level contacts between BITSNPRG57 and UCP1. (c–e) Violin plots showing distribution of (c) RMSD for Cα atoms and ligand, (d) RMSF for Cα atoms, and (e) RoG for UCP1–BITSNPRG57 complex. (f) Distribution of inter-residue distances between conserved arginine triplet reflecting conformational modulation in the presence of the ligand. (g–i) Time evolution plots of (g) RMSD, (h) RMSF, and (i) RoG capturing temporal structural stability and fluctuation. (j) DCCM highlighting correlated Cα fluctuations.

To probe whether these ligand-associated effects persist beyond the short time scale, the 100 ns trajectory was extended to 1 μs with continuous sampling. BITSNPRG57 remained confined to the central cavity throughout the extended simulation (Figure 9b) and retained contacts with residues on TM5/TM6, including Ser229 (TM5), Ile186, Leu277, and Trp280 (TM6), together with sustained engagement of the conserved arginine network. Among these, Ser229 and Trp280 contributed prominently to long-lived anchoring interactions. Notably, a stable direct H-bond between BITSNPRG57 and Ser229 emerged at ∼250 ns and persisted for the remainder of the trajectory, representing an interaction not previously reported for nucleotide- or DNP-bound UCP1 structures. After establishment of the Ser229 interaction, the ligand-fit protein RMSD showed reduced dispersion and approached a more stable regime (Figure 9c,g), consistent with a more settled binding pose within a relaxed cavity environment. Water-bridged contacts with Asp27/28 remained intermittent over the 1 μs trajectory, consistent with the BITSNPRG57’s preferred orientation deeper in the cavity and closer to Ser229.

Global stability metrics indicated that binding of BITSNPRG57 did not destabilize the protein. Cα RMSD showed a gradual transition without evidence of abrupt structural disruption, and RMSF profiles remained low across the protein (Figure 9d,h). The radius of gyration remained stable (≈20.2 Å; Figure 9e,i), consistent with an overall compact fold during the simulation. Distances within the conserved arginine network expanded gradually (Figure 9f), supporting a progressive relaxation of the central cavity geometry. Residue–residue distance and correlation analyses (Cα–Cα contact map/DCCM) preserved well-defined intradomain organization and persistent long-range packing (Figure 9j), indicating that cavity remodeling occurred without global unfolding. Taken together, these multimetric analyses support BITSNPRG57 as a structurally competent modulator of UCP1 cavity architecture over microsecond time scales. A schematic summary of its binding site placement relative to the conserved arginine network and Asp27/28 is shown in Figure 10.

**Figure 10:**
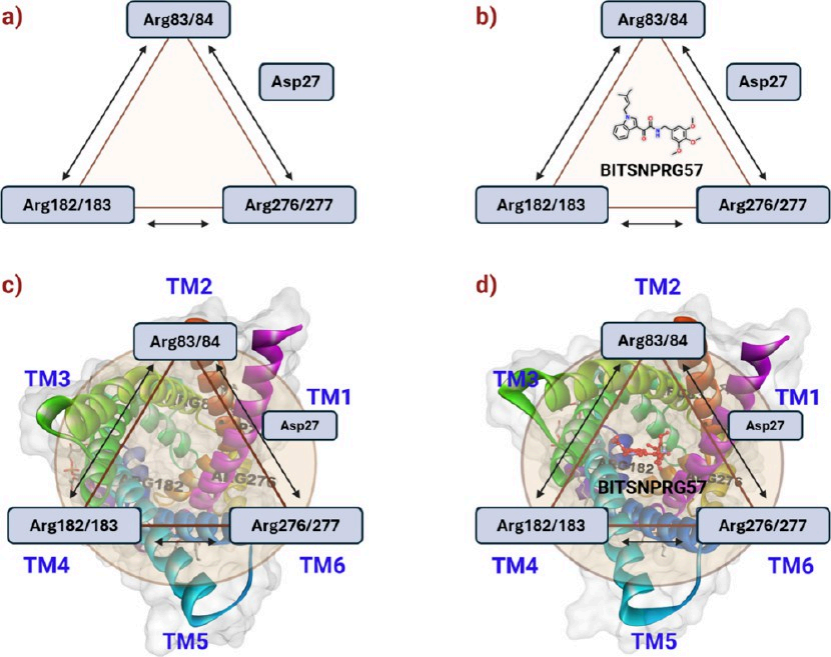
Spatial organization of the conserved arginine network, aspartic acid (D), and BITSNPRG57 in the UCP1 central cavity. (a) Conceptual schematic showing the triangular arrangement of the conserved arginine residues Arg83/84, Arg182/183, and Arg276/277, with Asp27/28 positioned proximal to the cavity. Double-headed arrows indicate the inter-residue distances monitored in the MD trajectories. (b) Schematic placement of BITSNPRG57 within the central cavity, illustrating its position relative to the arginine network and Asp27/28. (c) Structure-based view of the same residue set mapped onto the UCP1 transmembrane helices (TM1–TM6; color-coded as indicated). (d) Overlay highlighting the location of BITSNPRG57 (red) within the cavity in relation to the arginine network and Asp27/28. Created with BioRender.com.

Beyond UCP1, BITSNPRG57 has been reported as a potent PL inhibitor (IC_50_ = 4.15 μM).^39^ A previous structural analysis of its PL binding mode (PDB: 1LPB) reported engagement of catalytic-site residues, including an interaction with Ser152, consistent with α-ketoamide-mediated inhibition.^40^ Considered together with the present MD-derived UCP1 binding and cavity-modulation profile, BITSNPRG57 represents a plausible scaffold for further optimization toward dual-target activity. However, the present work is limited to atomistic simulations and does not directly establish UCP1-mediated proton conductance. Key determinants of functional activity, such as ligand protonation/ionization in the inner mitochondrial membrane environment and electrophysiological consequences, remain to be evaluated using dedicated computational and experimental assays.

## 3 Conclusion

This work presents an integrated computational analysis of how small-molecule analogues modulate the structure and dynamics of UCP1. Across atomistic MD simulations, the analogue set engaged conserved residues within the central cavity, most notably the conserved arginine network implicated in ligand recognition. Within this series, BITSNPRG57 showed the most persistent cavity occupancy and was associated with ligand-dependent relaxation of the arginine network geometry. This is consistent with the remodelling of the central cavity architecture relative to several comparator analogues and native ligands. Importantly, extended simulation of the BITSNPRG57–UCP1 complex (up to 1 μs) supported long-time scale stability of the bound state and revealed a sustained hydrogen-bond interaction with Ser229 (TM5), a contact not observed in available nucleotide- or DNP-bound reference structures. BITSNPRG57 also exhibited recurring water-mediated interactions involving Asp27/28, a residue previously linked to hydrated proton-handling motifs in UCP1. Together, these findings identify BITSNPRG57 as a promising scaffold for structure-guided optimization of UCP1-binding modulators. Considered alongside its reported PL inhibitory potency (IC_50_ = 4.15 μM), BITSNPRG57 provides a starting point for exploring dual-target antiobesity design strategies. While the present study is computational and does not directly quantify proton transport. However, the interaction and dynamics signatures defined here generate testable structural hypotheses to guide subsequent functional experiments and advanced simulation approaches aimed at validating UCP1-dependent uncoupling activity.

## 4 Computational Details

### 4.1 Data Curation

The chemical analogues used in this investigation were selected from an in-house library of antiobesity agents (PL inhibitors). These analogues underwent prefiltering to exclude less potent candidates, applying an IC_50_ threshold of <5 μM against PL. Data curation and property-based filtering were done using the DataWarrior software, facilitating efficient processing, clustering, and selection of optimal candidates. Property clustering was performed using a modified LogP/intestinal absorption filter from our recent report.^24^

### 4.2 Ligand Structure Preparation

Chemical structures were generated in ChemDraw Professional 23.1.1 (Revvity Signals Software, Inc.) and converted to three-dimensional geometries. Initial molecular mechanics minimization was performed using the MMFF94 force field (steepest descent) in Avogadro (ORCA-enabled build).^41,42^

### 4.3 Quantum Chemistry Computation Using the DFT Method

Geometry optimization and electronic structure calculations were carried out in ORCA 5.0.4, following previously reported procedures.^43^ Input files for the calculations were prepared in ORCA, and the output files were analyzed using orca-enhanced Avogadro.^44^ All ligands were optimized in the gas phase using B3LYP/def2-TZVP with RIJCOSX acceleration and D4 dispersion correction (OPT RIJCOSX D4).^45–49^ The conductor-like polarizable continuum model (CPCM) was used for solvent phase calculations. Frontier molecular orbital (FMO) energies were computed at the same level of theory, and global reactivity descriptors were derived from FMO energies using Koopmans-based expressions.^50^

### 4.4 Protein–Ligand Complex Preparation (by Docking)

The protein–ligand complexes were generated using docking. Human UCP1 cryo-EM structures (apo: 8HBV; DNP: 8J1N; ATP: 8HBW; GTP: 8G8W; UTP: 9FZQ) were obtained from the RCSB Protein Data Bank.^12,25,26^ Protein preparation was performed in Maestro (Schrödinger) using the Protein Preparation Wizard/Prime: bond orders were assigned, missing hydrogens were added, and protonation states were generated (state method and pH, e.g., Epik at pH 7.0 ± 2.0). Nonprotein components used for structure determination (e.g., nanobodies/sybodies) were removed. Structurally resolved bound lipids (including cardiolipin and phosphatidylcholine) were retained where present to preserve native-like lipid–protein contacts.^51^ A previously validated docking protocol using the same computational system and protein structures was followed.^24^ Docking was performed using Glide in extra-precision (XP) mode.^52^ A receptor grid was generated for the UCP1 central cavity and centered on the cocrystallized ligand binding region. For each analogue, up to 10 poses were generated using default XP settings unless otherwise noted.

#### 4.4.1 Selection of Pose for MD Simulation

For each ligand, the docked pose selected for MD was chosen based on (i) a plausible interaction fingerprint within the UCP1 central cavity (including conserved cavity residues) and (ii) Prime MM-GBSA rescoring (Maestro (Schrödinger); OPLS2005 force field; VSGB 2.0 implicit solvent).^53,54^ The pose exhibiting the most favorable MM-GBSA profile, together with consistent cavity engagement, was advanced to MD

### 4.5 Molecular Dynamics Simulation

The academic version of Schrödinger Desmond software, integrated within Maestro, was utilized for complex system building, energy minimization, MD production runs, and simulation interaction analysis for all complexes.

#### 4.5.1 Setting Up Protein–Ligand Systems for Simulation

Protein–ligand complexes prepared via the docking protocol were used for MD simulation. The Desmond System Builder module was used to assemble these complexes. To closely mimic the native membrane environment of UCP1, the complexes were embedded within a pre-equilibrated palmitoyl-oleoyl-phosphatidylcholine (POPC) lipid bilayer.^55^ The UCP1 construct comprising residues 5–295, encompassing all six TM helices, was used for membrane insertion. Experimentally resolved cardiolipin and phosphatidylcholine molecules present in the cryo-EM structure were retained to preserve native lipid–protein interactions. The membrane system consisted of 86 POPC lipid molecules distributed symmetrically across the bilayer (43 lipids per leaflet). All simulated systems contained POPC lipids and were generated using the System Builder module of the Schrödinger software suite. POPC was chosen as a widely accepted and computationally efficient bilayer model, and it has been shown to support the native-like conformation and activity of mitochondrial IMM proteins.^56^ Protein protonation states were assigned in Maestro at physiological pH (∼7.4), with manual inspection of histidine tautomers. Each system was solvated using TIP3P water molecules in an orthorhombic simulation box with a 10 Å buffer distance and neutralized with Na^+^/Cl^–^ ions to achieve a physiological salt concentration of 0.15 M NaCl. Periodic boundary conditions were applied using a water box of dimensions 10 Å × 10 Å × 10 Å. All systems were energy-minimized and equilibrated using Desmond’s default protocol prior to production MD simulations with the OPLS2005 force field.

#### 4.5.2 Equilibration and Production Runs

The protein–ligand complexes were equilibrated and subjected to production MD simulations under the isothermal–isobaric (*NPT*) ensemble. During *NPT* equilibration, the system pressure was maintained at 1.01325 bar using the Martyna–Tobias–Klein (MTK) barostat to ensure stable pressure during temperature fluctuations. Simulations employed the OPLS2005 force field with a 2 fs time step and the RESPA integrator. Temperature and pressure were controlled at 310.15 K and 1.01325 bar, respectively, using the Nose–Hoover thermostat (relaxation time: 1 ps) and the MTK barostat (relaxation time: 5 ps).^57^ Long-range electrostatic interactions were calculated using the particle-mesh Ewald method with a cutoff radius of 12 Å for consistent interaction modeling.^58^ The final production run for each complex was conducted for 100 ns, generating 2000 trajectory frames for detailed analysis. Table S2 outlines the simulation parameters and descriptors used to evaluate structural stability, flexibility, intermolecular interactions, energetic behavior, dynamic properties, solvent exposure, and statistical distributions of the molecular systems.

## Supplementary Material





## Data Availability

All data supporting the findings presented in this study are included in this Main Draft and its Supporting Information files.

## Notes

Information related to DFT study; Figure S1: docking-related data; Figure S2: DFT-related data; Figure S3–S13: superimposed view and RMSD for optimized geometry from DFT; Figure S14–S24: structural positioning; S25–S39 MD: trajectory analysis; Table S1: calculated energies; Table S2: parameters investigated in post-MD simulation analysis (PDF)
Video S1: MD trajectory illustrating the interactions shown in Figure 11 (MP4)
