# Supplementary material for: Interactions and Conformational Plasticity of Human Uncoupling Protein 1 in Response to Small-Molecule Analogues: Insights from Molecular Dynamics Simulations
Source: ACS Omega. 2026 Jun 11;11(24):35503–17. doi: 10.1021/acsomega.6c01124 (PMC13295038; doi:10.1021/acsomega.6c01124)
Supplement: Supplementary file 1 [file ao6c01124_si_001.pdf]

Supplementary Information

Interactions and conformational plasticity of human uncoupling protein 1 in response to small molecule analogues: Insights from molecular dynamics simulations

Sanket Rathod[1,2#], Utkarsh A. Jagtap[1#], Falguni Pankhania[1,3], Ravi Shukla[2,4], Atish T. Paul[1]*

[1]Laboratory of Natural Product Chemistry, Department of Pharmacy, Birla Institute of Technology and Science, Pilani, Pilani Campus, Vidya Vihar, Pilani, Rajasthan 333031, India.

[2]School of Science, RMIT University, Melbourne, VIC, 3000, Australia.

[3]Biomedical Research, PK Sciences, Novartis Healthcare Private Limited, Hyderabad, Telangana 500032, India.

[4]NanoBiotechnology Research Laboratory, Centre for Advanced Materials & Industrial Chemistry, RMIT University, Melbourne, VIC, 3001, Australia.

[#]*These authors contributed equally*

Graphical abstract

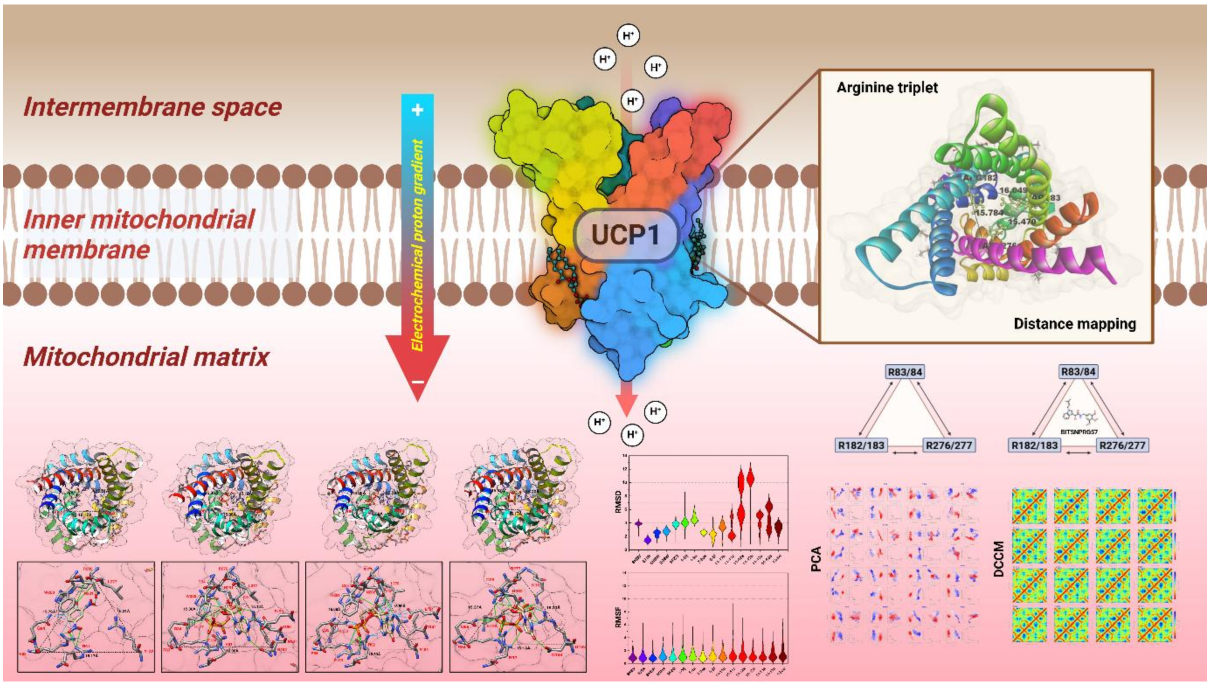

DFT study

All DFT calculations were performed in the gas phase using ORCA, and the outputs were analyzed using the ORCA-enhanced Avogadro interface. The B3LYP functional with the DEF2-TZVP basis set was selected for its well-documented accuracy in predicting structural and electronic properties of drug-like molecules. As solvation effects were not included in this study, correlation with biological activity was not attempted; instead, structural and reactivity insights were prioritized. DFT calculations served two major purposes in the current investigation. First, they aimed to optimize the geometries of the 11 shortlisted analogues' chemical structures to generate reliable conformers for protein-ligand docking (protein-ligand systems preparation) and subsequent MD simulations. For the GO of the selected analogues, the OPT RIJCOSX D4 method was employed.[1,2] This approach uses the Resolution of Identity (RI) technique with the JCOSX approximation to improve computational efficiency. Along with RIJCOSX, the D4 dispersion correction is used to accurately account for non-covalent interactions and enhance the precision of the result. The second objective was to perform a chemoinformatic analysis to assess the electronic properties and chemical reactivity of the selected analogues.

GO was performed for all 11 analogues (structure provided in **Figure 4**), and the optimized structures were used for constructing protein-ligand complexes for MD simulations. As a result of GO, significant structural differences were observed between the pre- and post-optimized conformations for some analogues. It was considered that the structures exhibited chemically valid conformations, as evidenced by bond lengths and angles, in accordance with quantum mechanical principles. Additionally, the conformational change suggests that the analogues have possibly approached the energy-minimized states. These findings highlight the importance of proper GO of the chemical structures, as the initial sketched structures may not represent realistic chemical conformations. Previous studies show that DFT-based optimization can predict molecular geometries that closely resemble experimentally resolved structures, such as those obtained by single-crystal X-ray diffraction.[3] This approach is conceptually analogous to homology modelling in protein structure prediction, where the goal is to model and mimic experimentally determined protein conformations. Similarly, through GO, we aimed to achieve chemically accurate and experimentally relevant geometries of small molecules, thereby providing a reliable foundation for subsequent computational analyses. For this purpose, FMO analysis and the computation of quantum chemical descriptors (QCDs) were performed. This was done to get insights into the chemical profile of the analogues under investigation. The data of the evaluated parameters are summarized in **Table S1**. Additionally, the spatial localization and energy levels of HOMO and LUMO orbitals, along with E_{HLgap}, are illustrated in **Figure S2**.

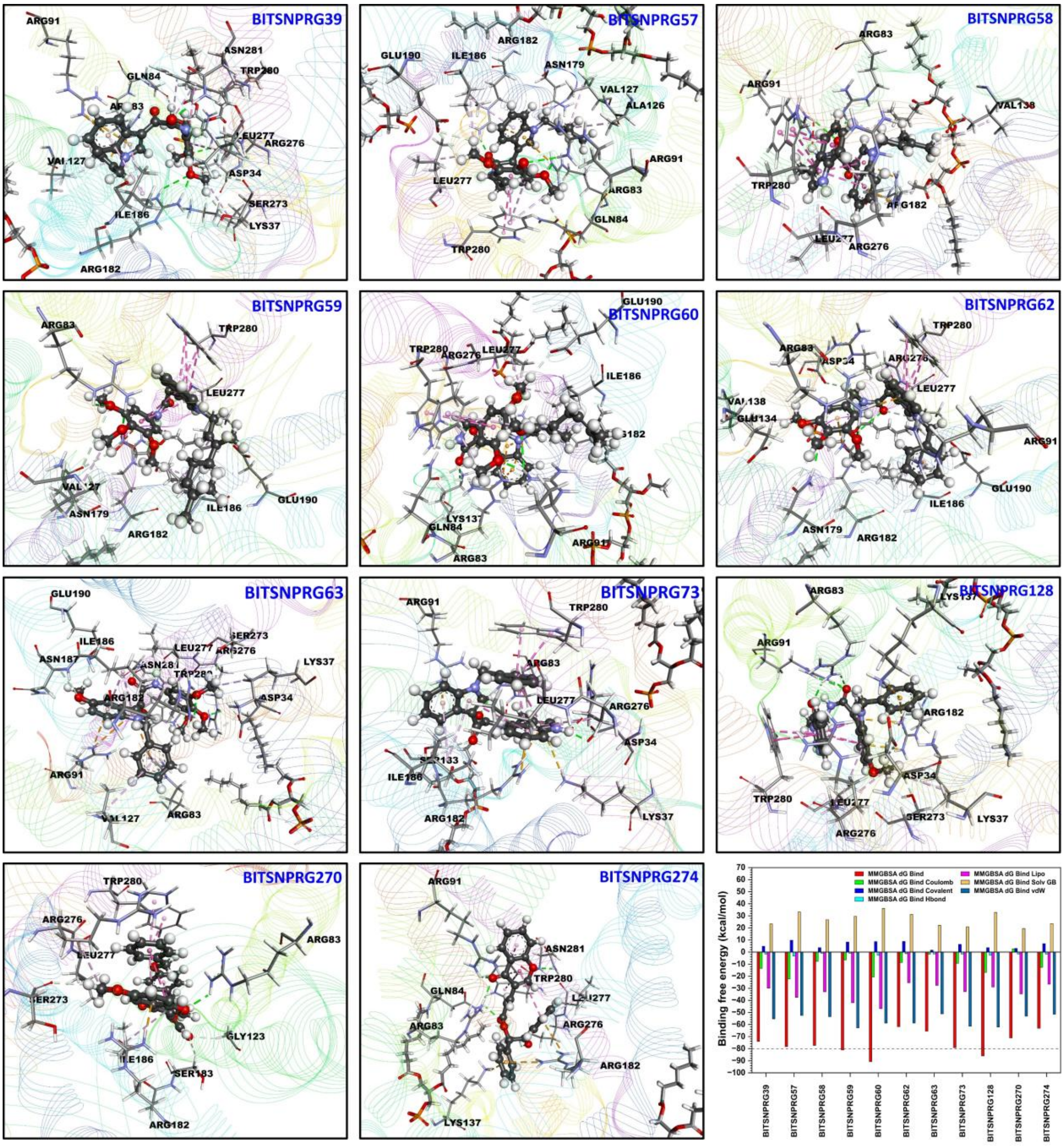

Figure S1. **Binding pose analysis and interaction profiling of in-house chemical analogues with UCP1**. Panels depict the molecular interactions of selected analogues docked into the substrate binding site of UCP1 (PDB: 8J1N). Highlighting key residues involved in ligand recognition, hydrogen bonding, hydrophobic contacts, and polar interactions. Each subpanel illustrates the docked binding orientation of an individual analogue (labelled top right), surrounded by key residues within the binding cavity; **a**) Bar graph showing calculated profile of MMGBSA binding free energies for the selected docked complex (data for the other nine conformers not presented).

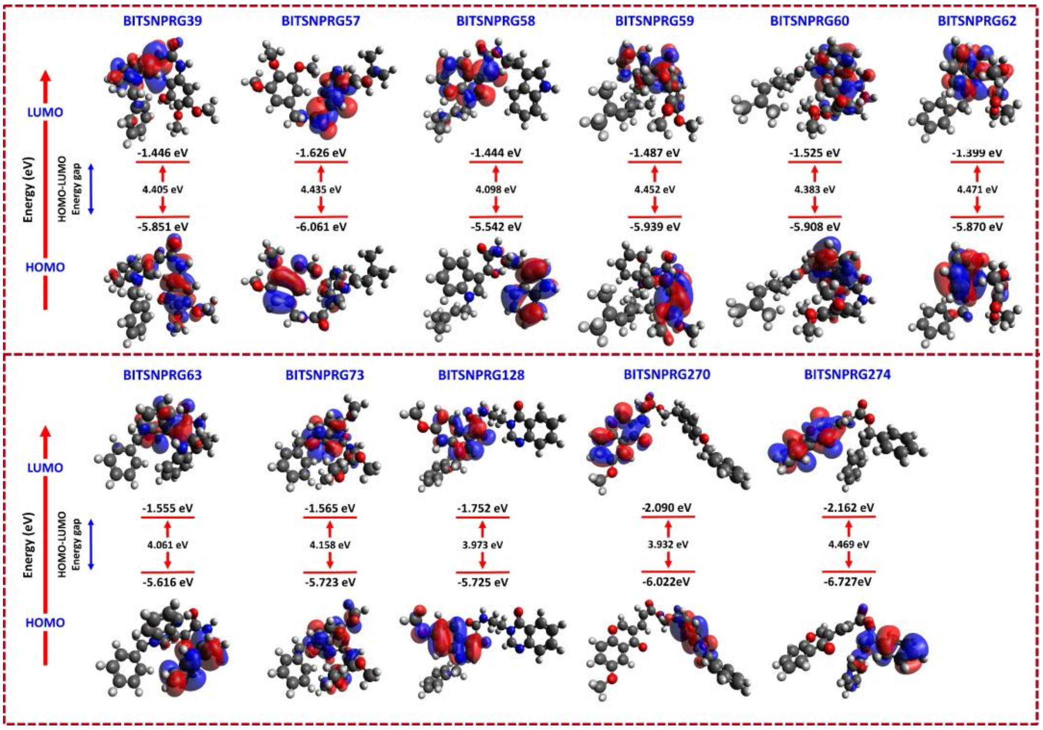

Figure S2. FMO localization along with energies of HOMO (E_{HOMO}), LUMO (E_{LUMO}), and HOMO-LUMO energy gap (E_{HLgap}). The isosurface representations depict the spatial distribution of molecular orbitals, with blue and red lobes denoting opposite phases of the wavefunction.

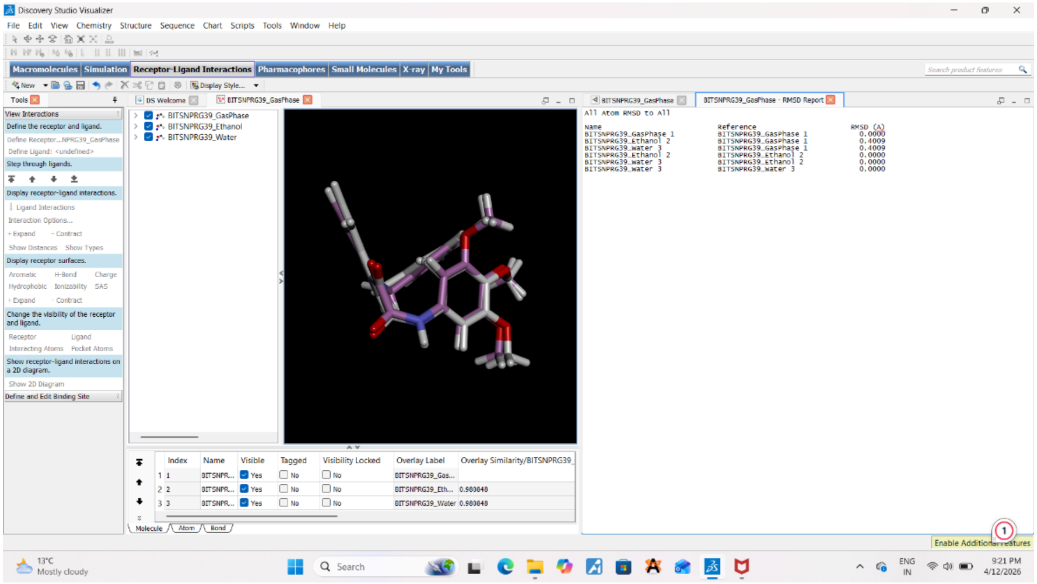

Figure S3. Superimposed view and RMSD for optimized geometry in Gas, water, and ethanol for BITSNPRG39

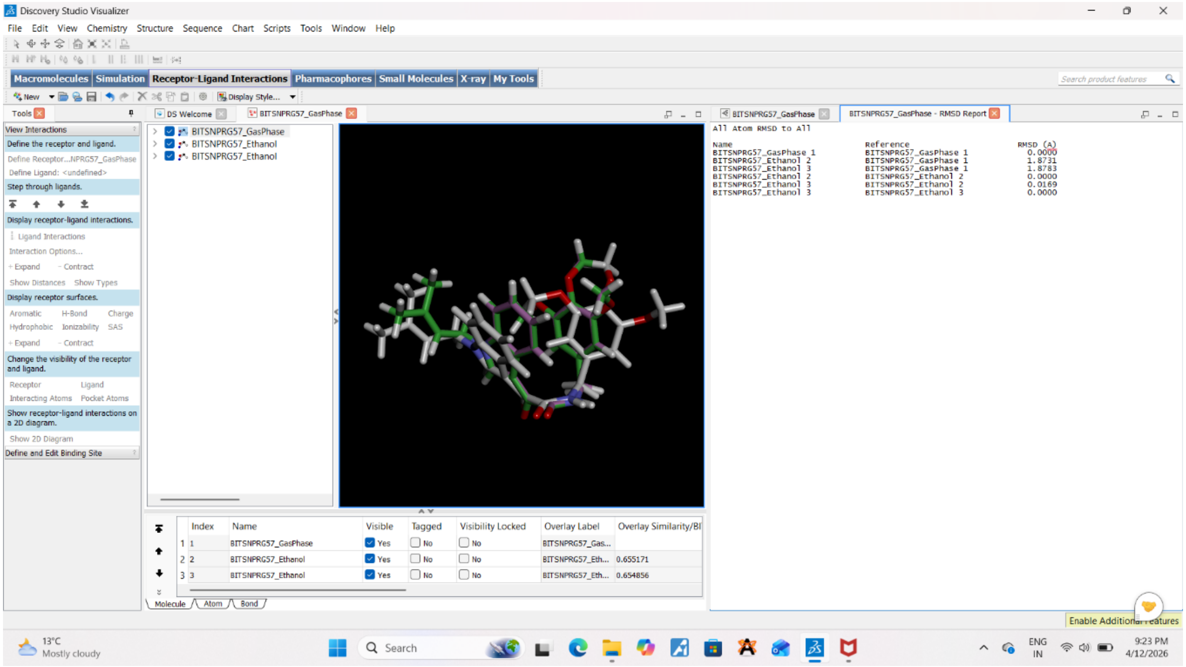

Figure S4. Superimposed view and RMSD for optimized geometry in Gas, water, and ethanol for BITSNPRG57

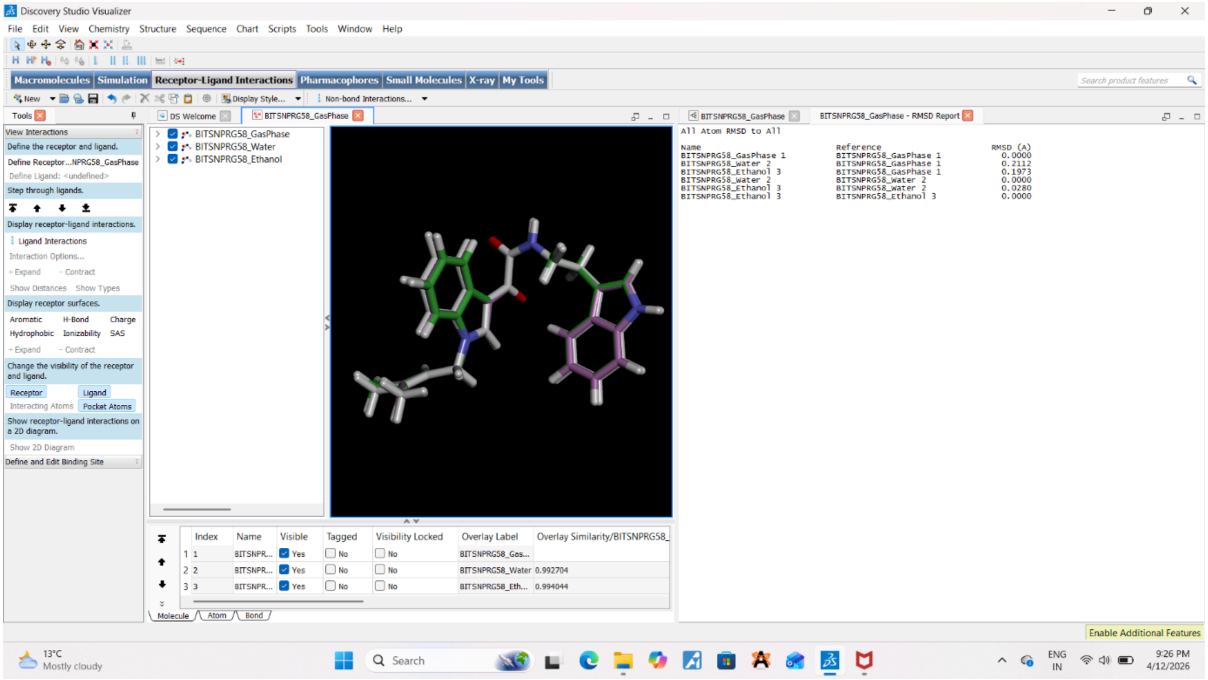

Figure S5. Superimposed view and RMSD for optimized geometry in Gas, water, and ethanol for BITSNPRG58

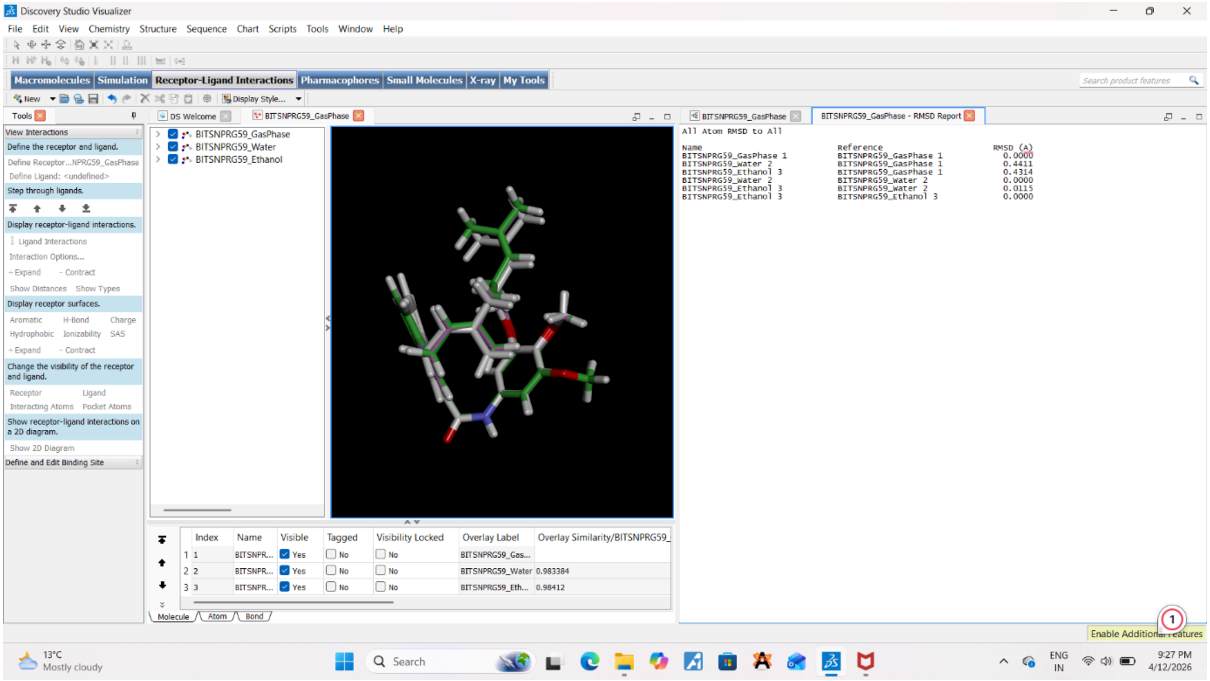

Figure S6. Superimposed view and RMSD for optimized geometry in Gas, water, and ethanol for BITSNPRG59

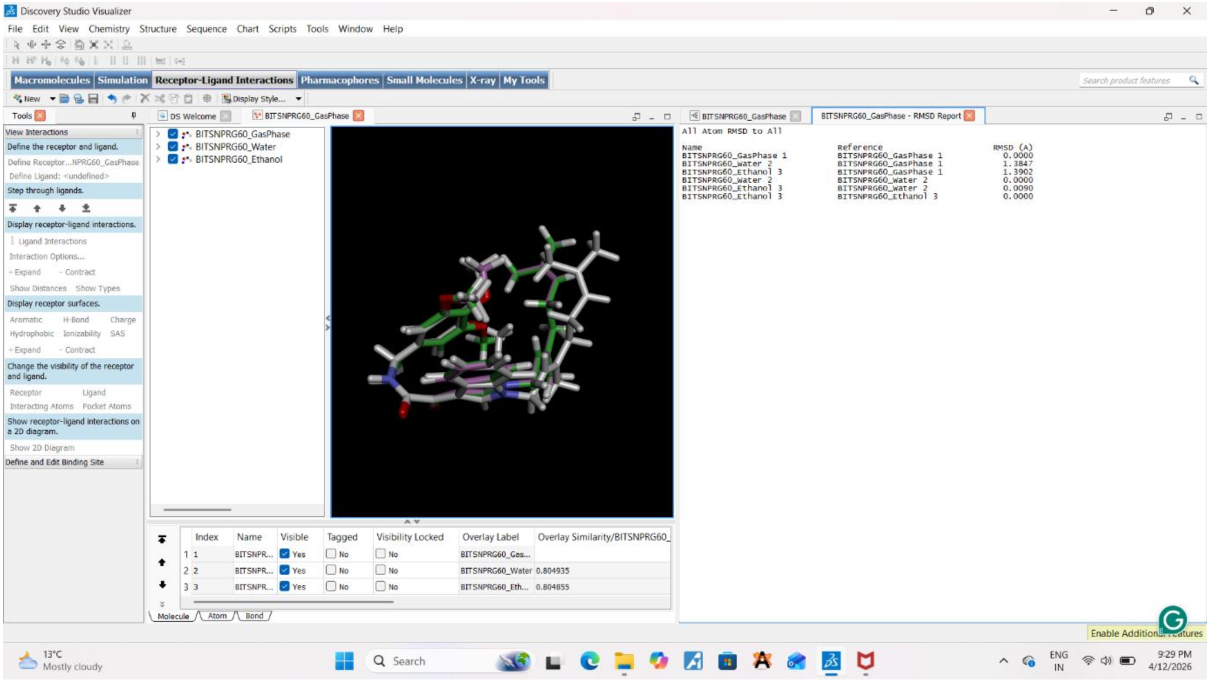

Figure S7. Superimposed view and RMSD for optimized geometry in Gas, water, and ethanol for BITSNPRG60

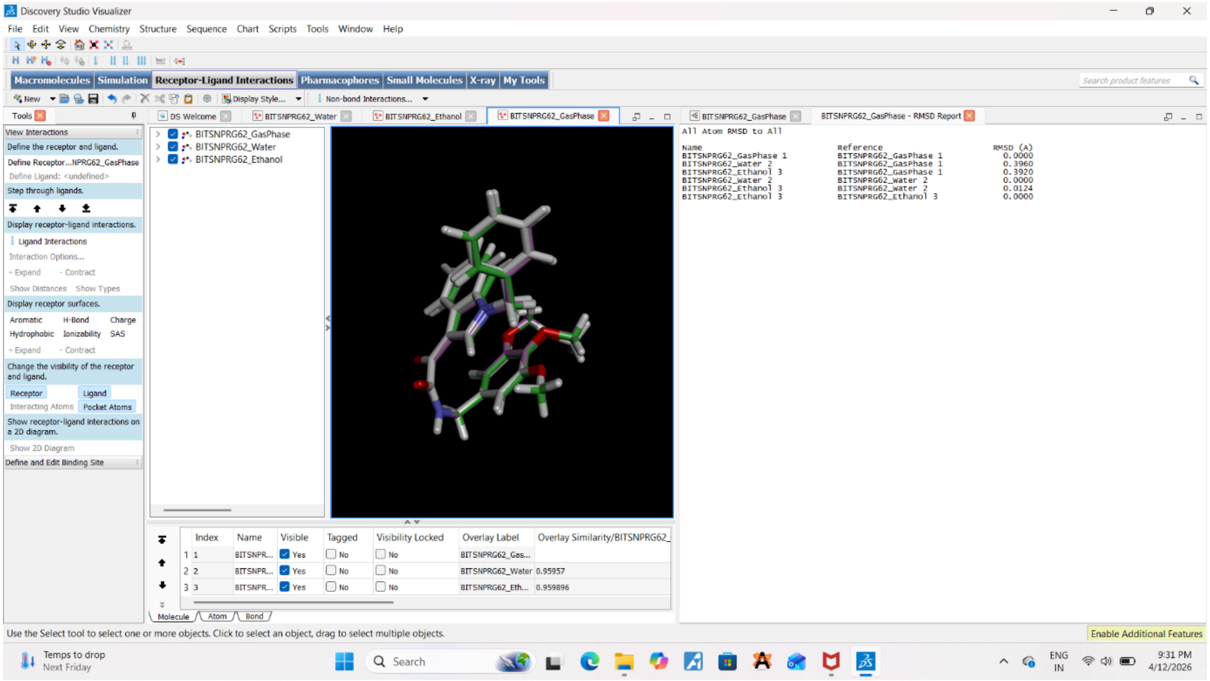

Figure S8. Superimposed view and RMSD for optimized geometry in Gas, water, and ethanol for BITSNPRG62

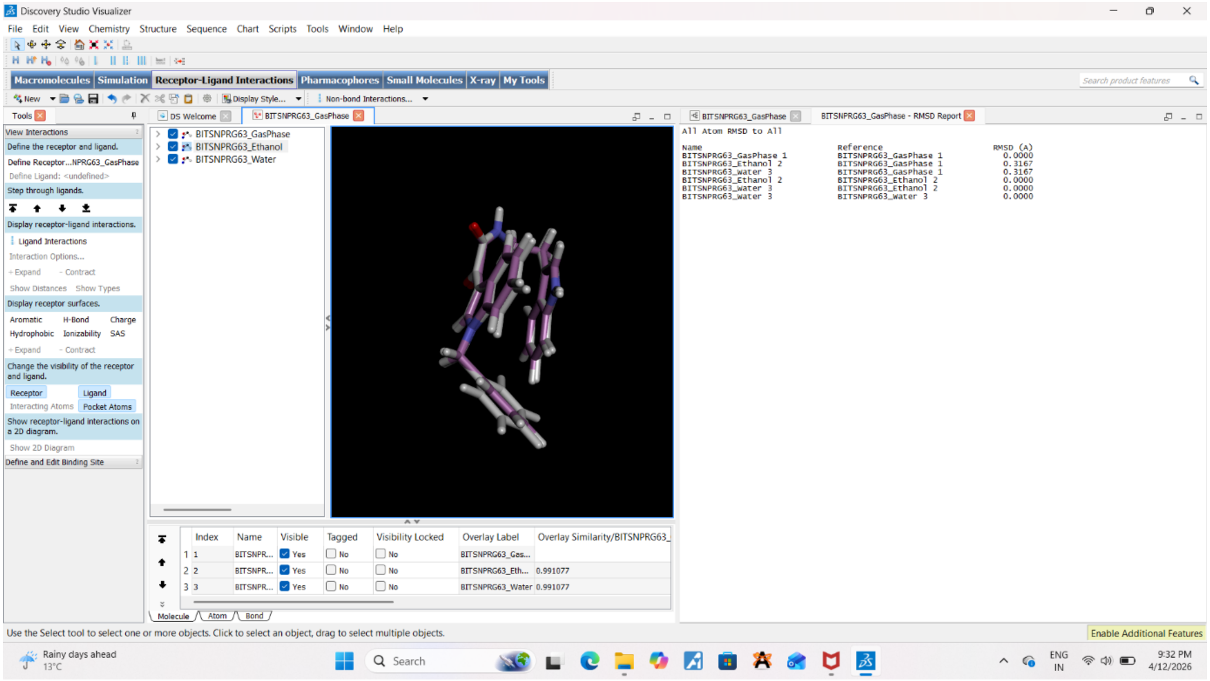

Figure S9. Superimposed view and RMSD for optimized geometry in Gas, water, and ethanol for BITSNPRG63

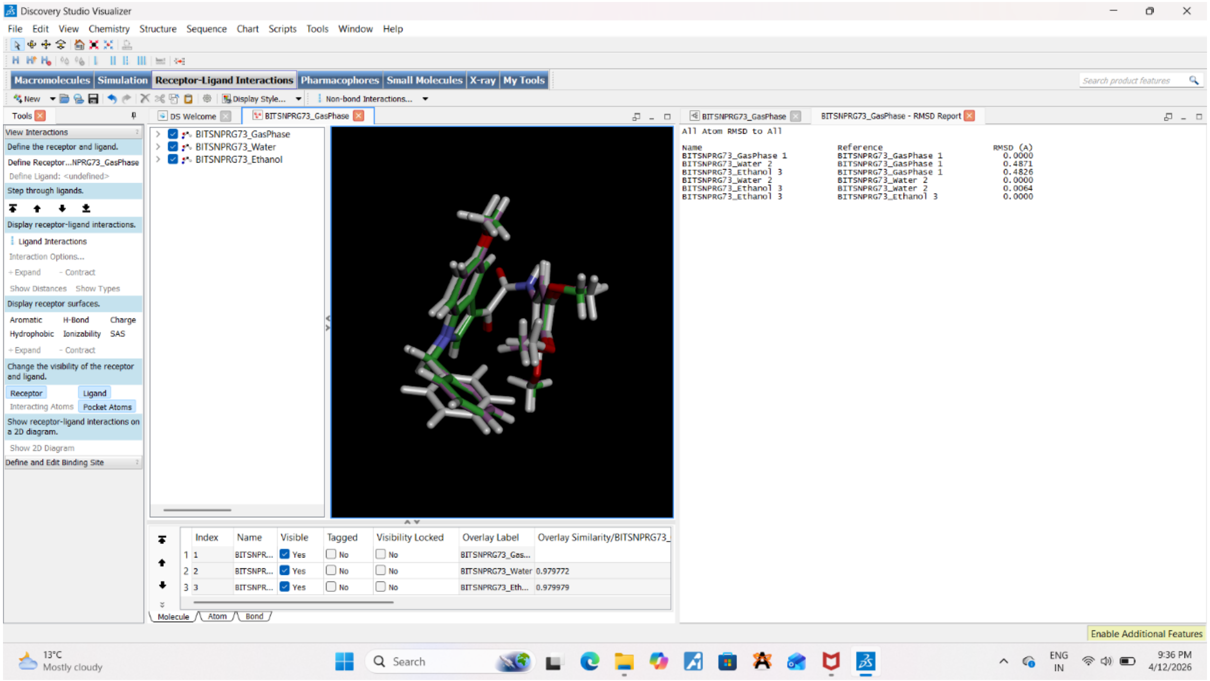

Figure S10. Superimposed view and RMSD for optimized geometry in Gas, water, and ethanol for BITSNPRG73

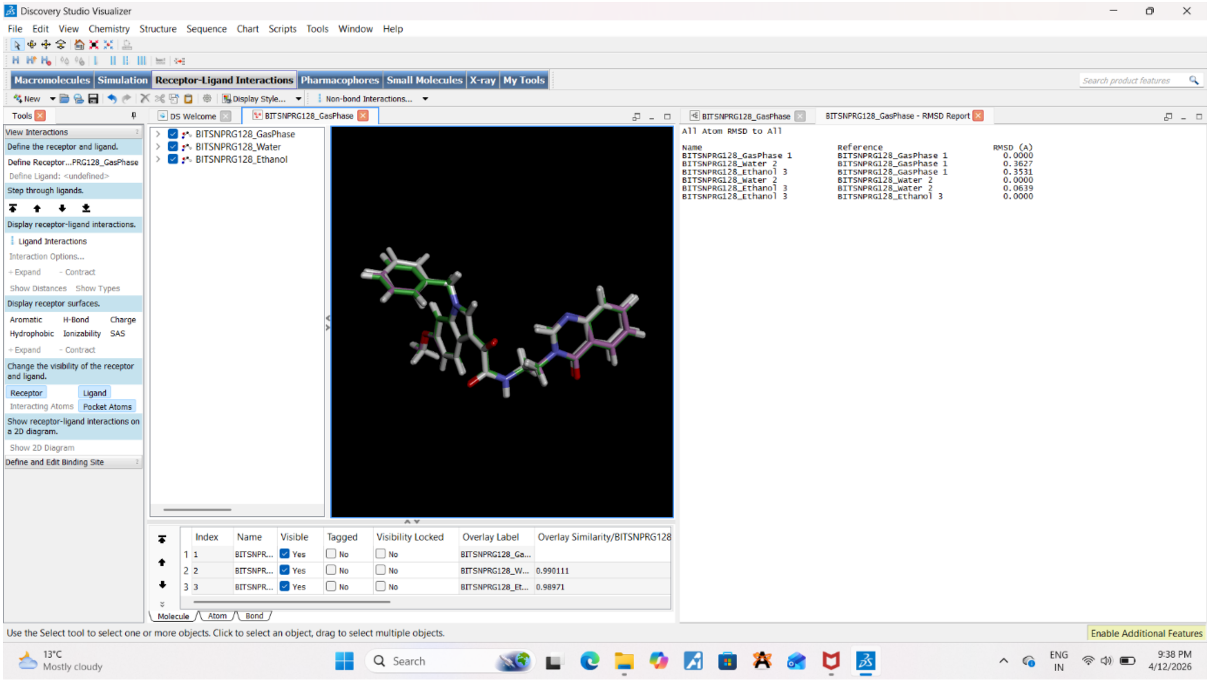

Figure S11. Superimposed view and RMSD for optimized geometry in Gas, water, and ethanol for BITSNPRG128

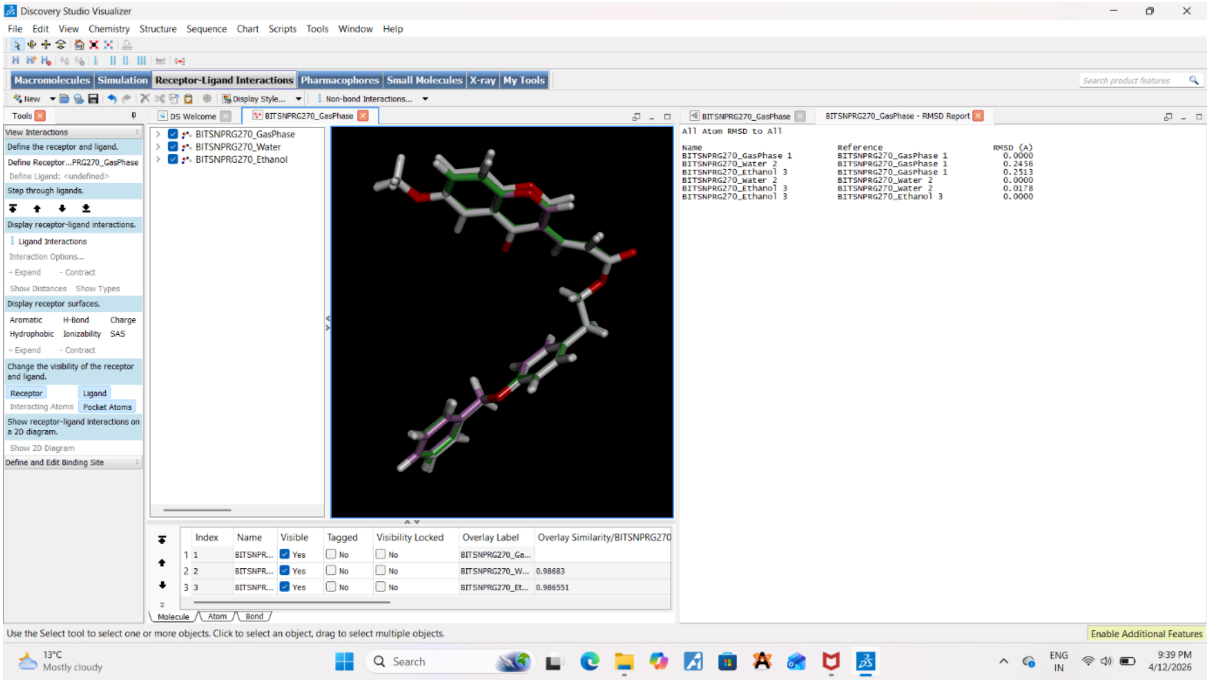

Figure S12. Superimposed view and RMSD for optimized geometry in Gas, water, and ethanol for BITSNPRG270

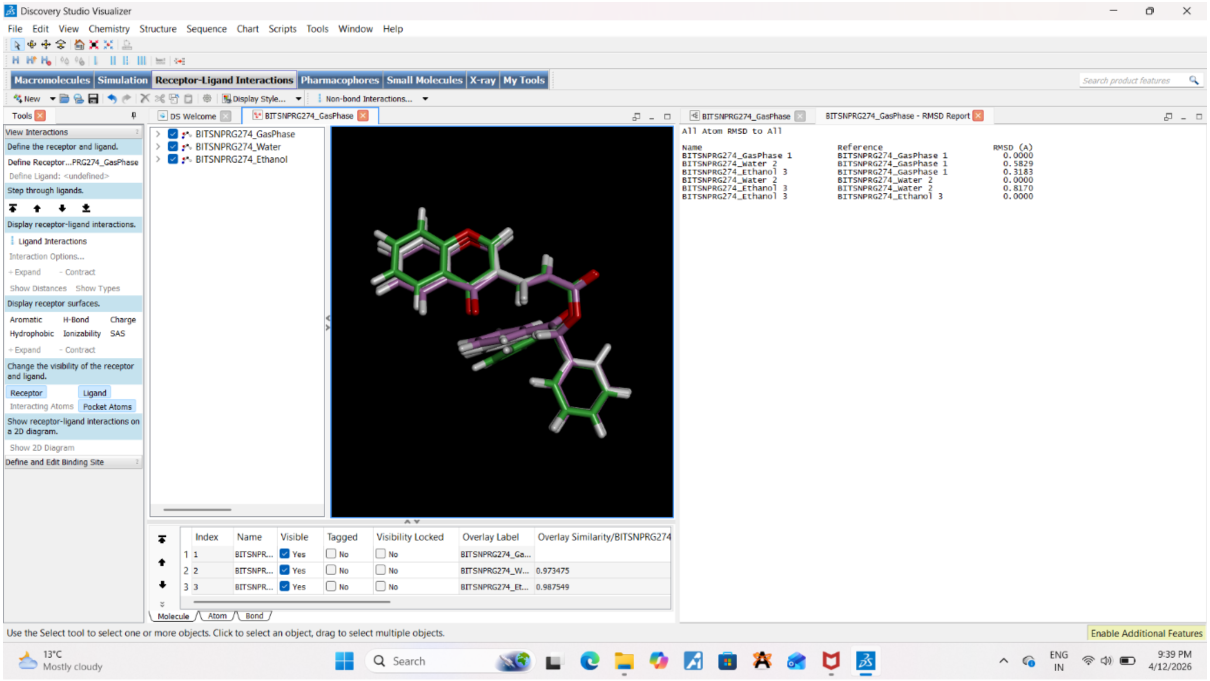

Figure S13. Superimposed view and RMSD for optimized geometry in Gas, water, and ethanol for BITSNPRG274

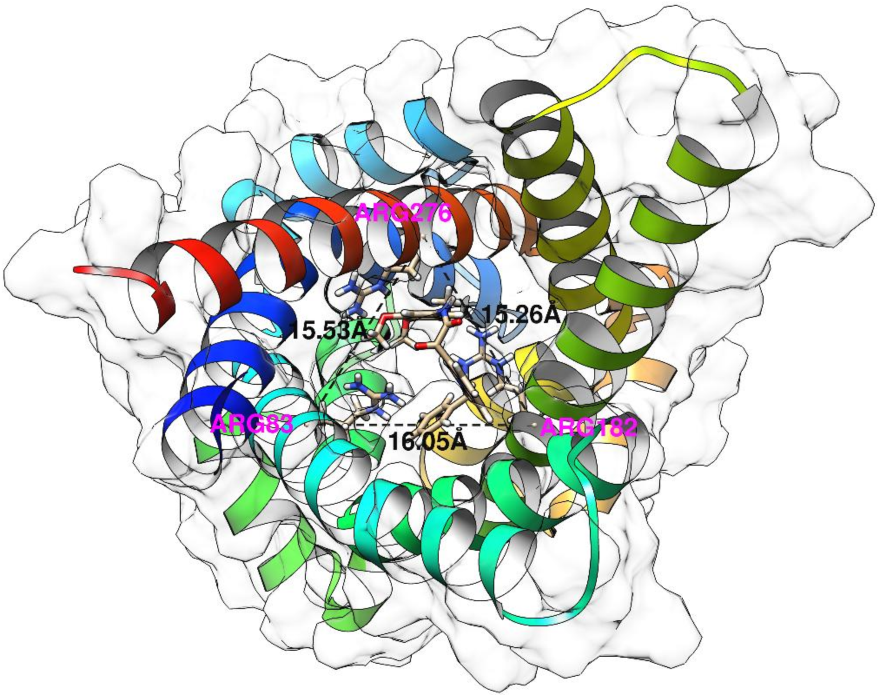

Figure S14. Structural positioning of BITSNPRG39 within the central cavity of UCP1. In-house chemical analogues (shown in tan) docked into the central binding cavity of UCP1. Dotted lines indicate the spatial arrangement and inter-residue distances between the conserved arginine triplet (Arg83/84, Arg182/183, Arg276/277), highlighting ligand-induced modulation of cavity geometry. Numerical values adjacent to each dotted line represent the corresponding Cα–Cα distances (in Å).

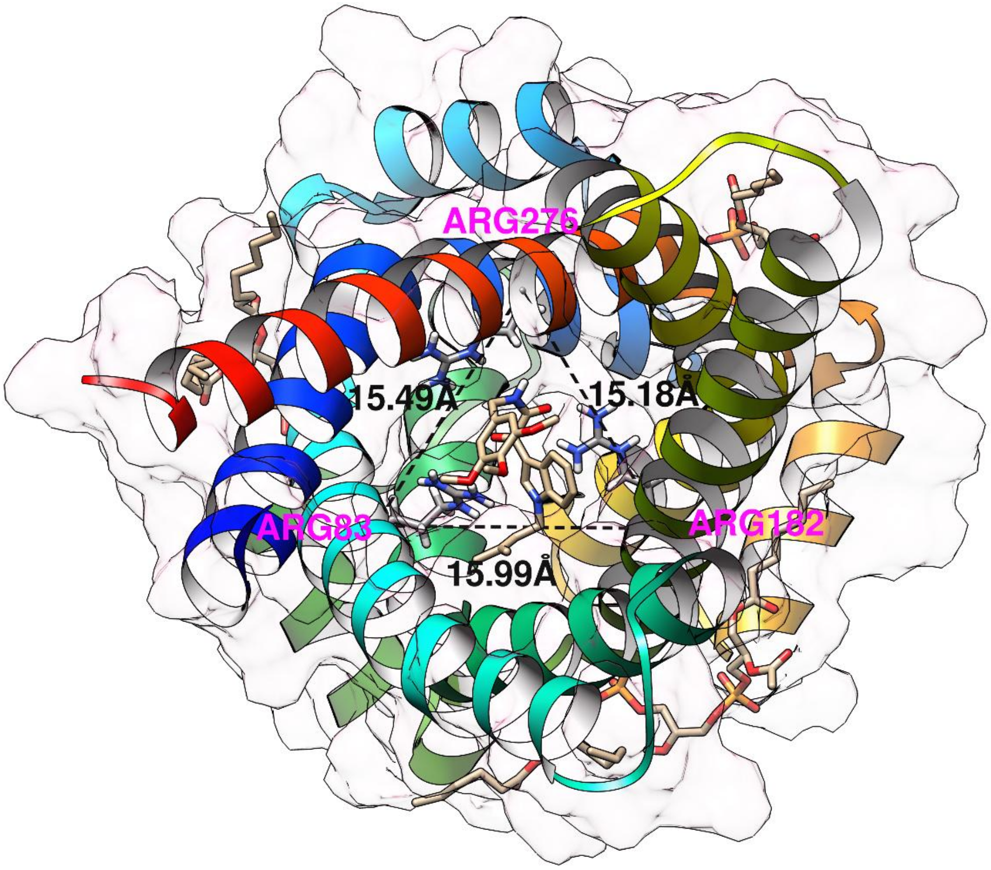

Figure S15. Structural positioning of BITSNPRG57 within the central cavity of UCP1

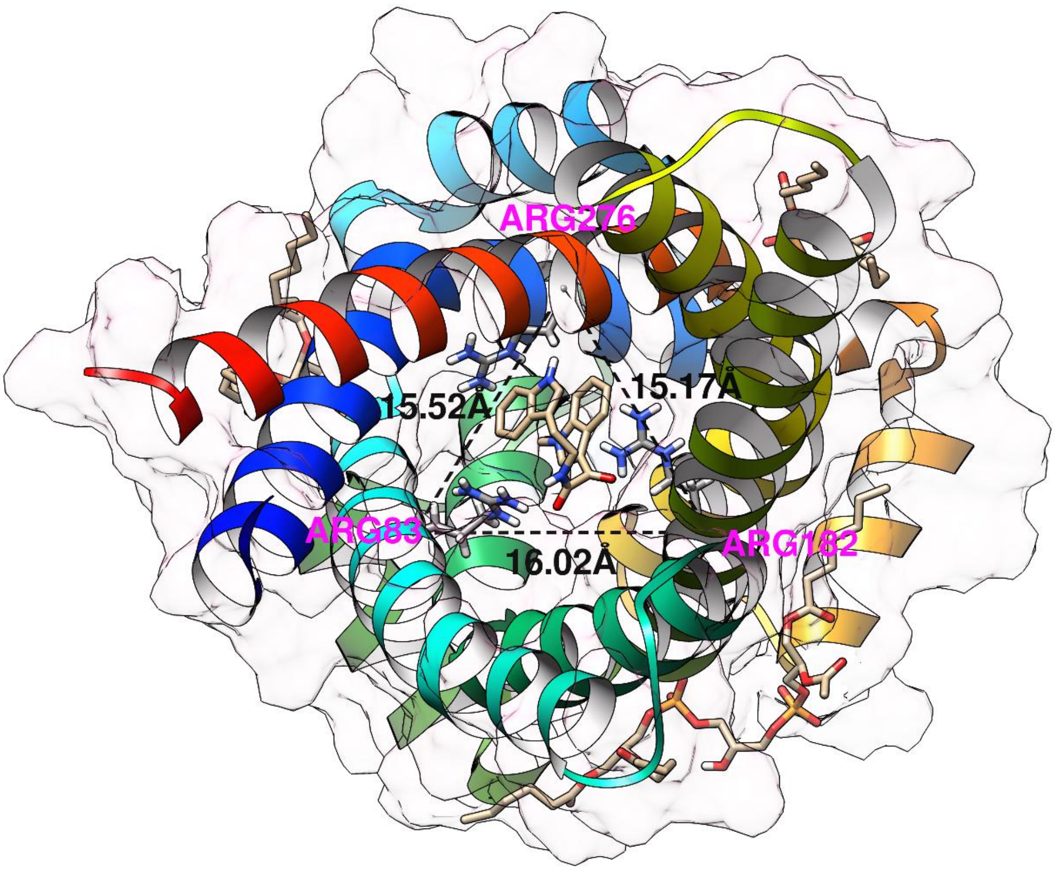

Figure S16. Structural positioning of BITSNPRG58 within the central cavity of UCP1

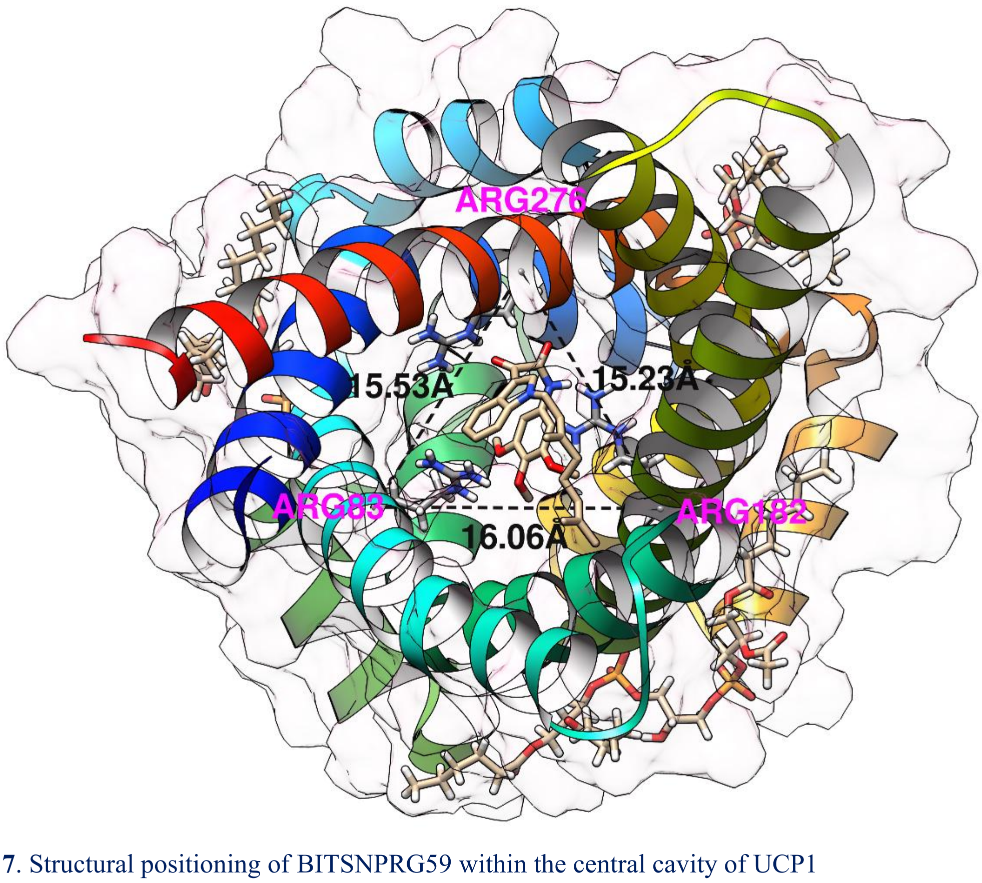

Figure S17. Structural positioning of BITSNPRG59 within the central cavity of UCP1

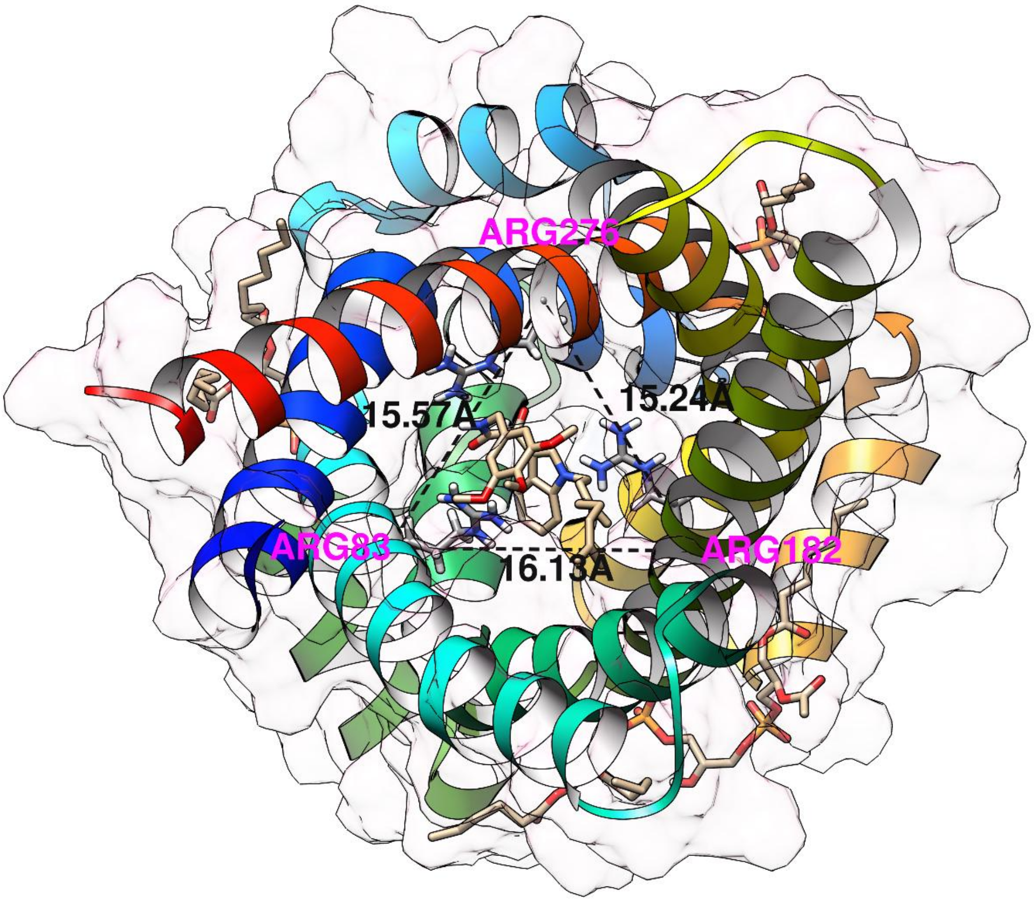

Figure S18. Structural positioning of BITSNPRG60 within the central cavity of UCP1

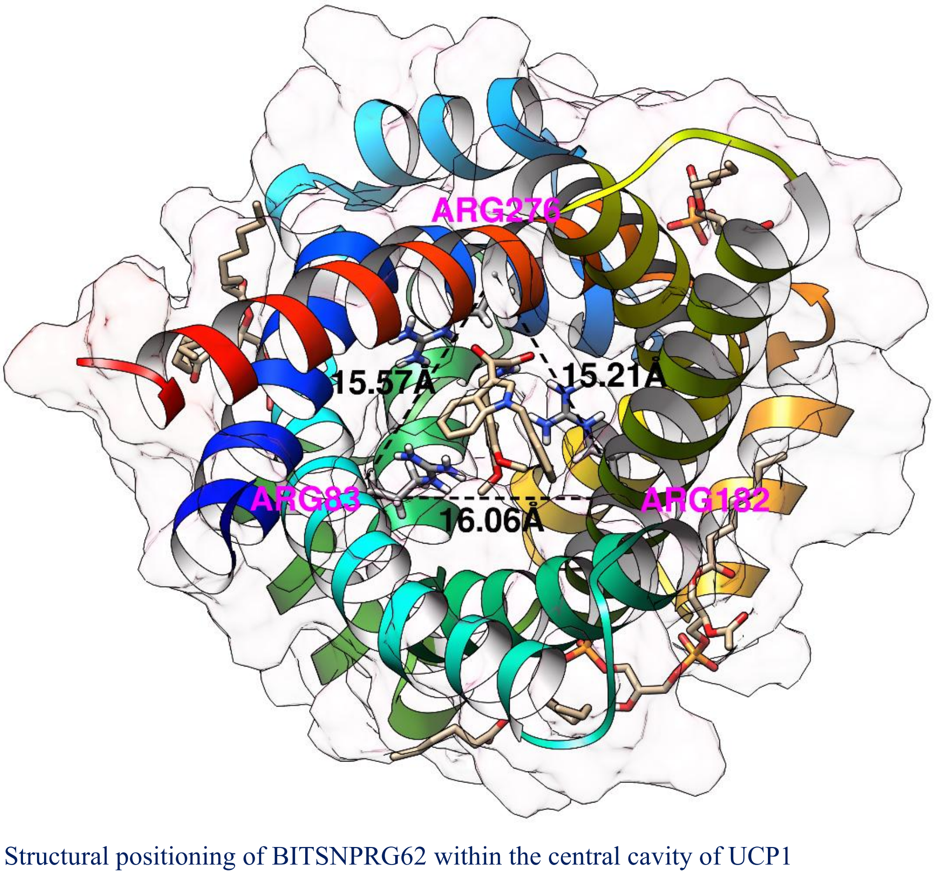

Figure S19. Structural positioning of BITSNPRG62 within the central cavity of UCP1

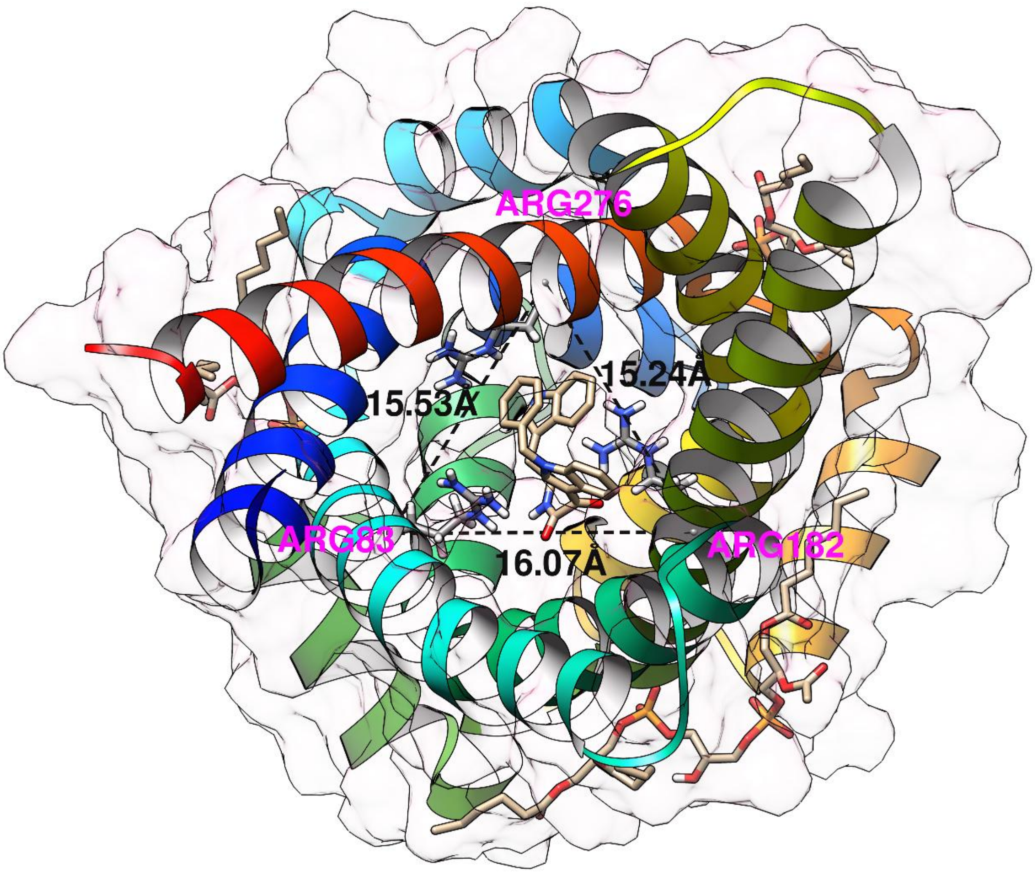

Figure S20. Structural positioning of BITSNPRG63 within the central cavity of UCP1

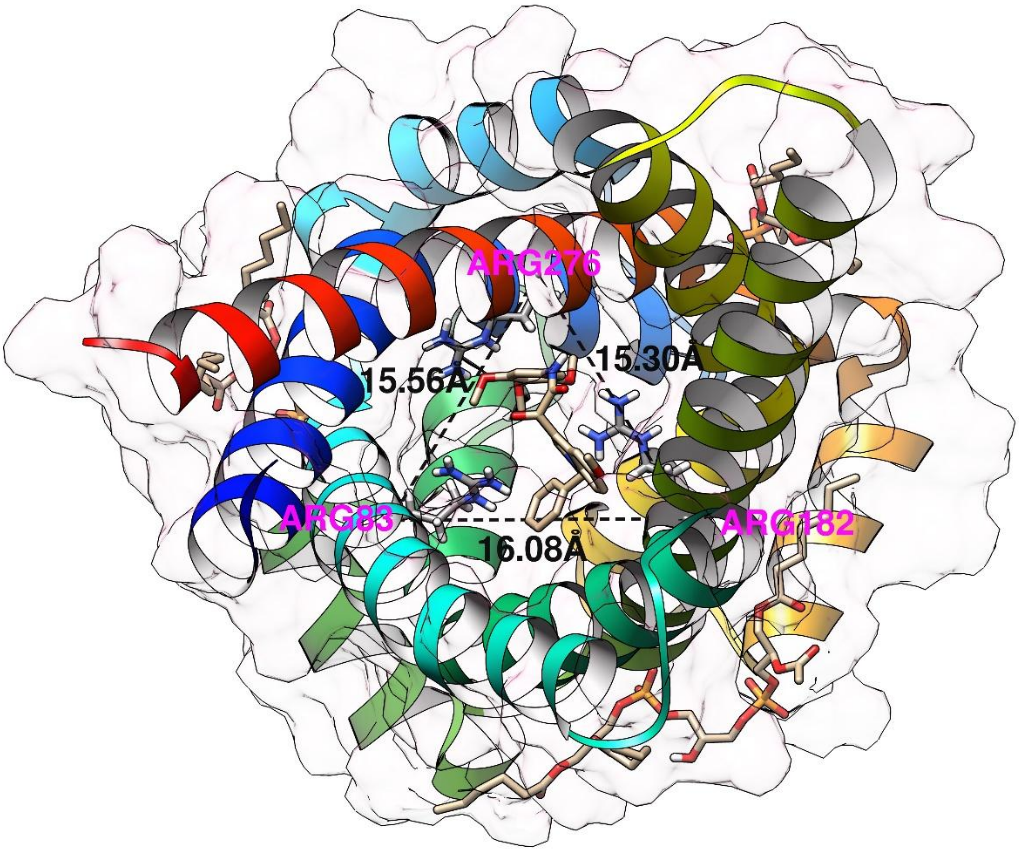

Figure S21. Structural positioning of BITSNPRG73 within the central cavity of UCP1

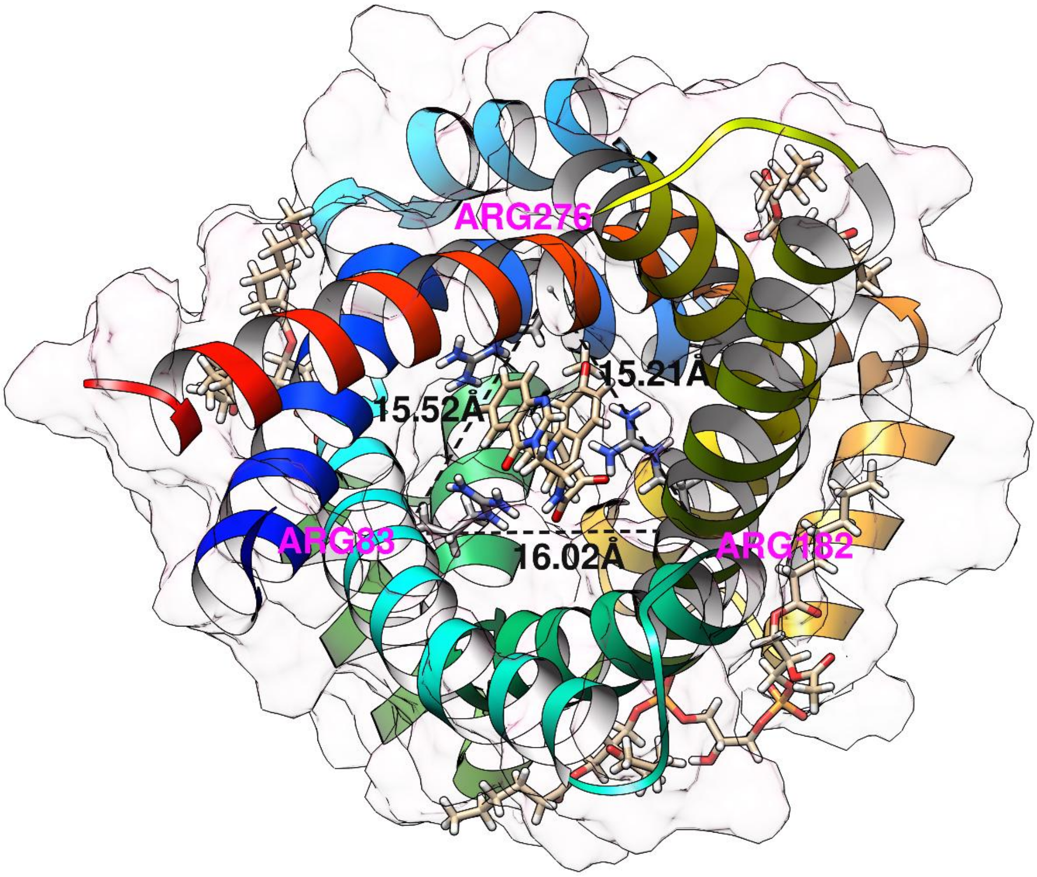

Figure S22. Structural positioning of BITSNPRG128 within the central cavity of UCP1

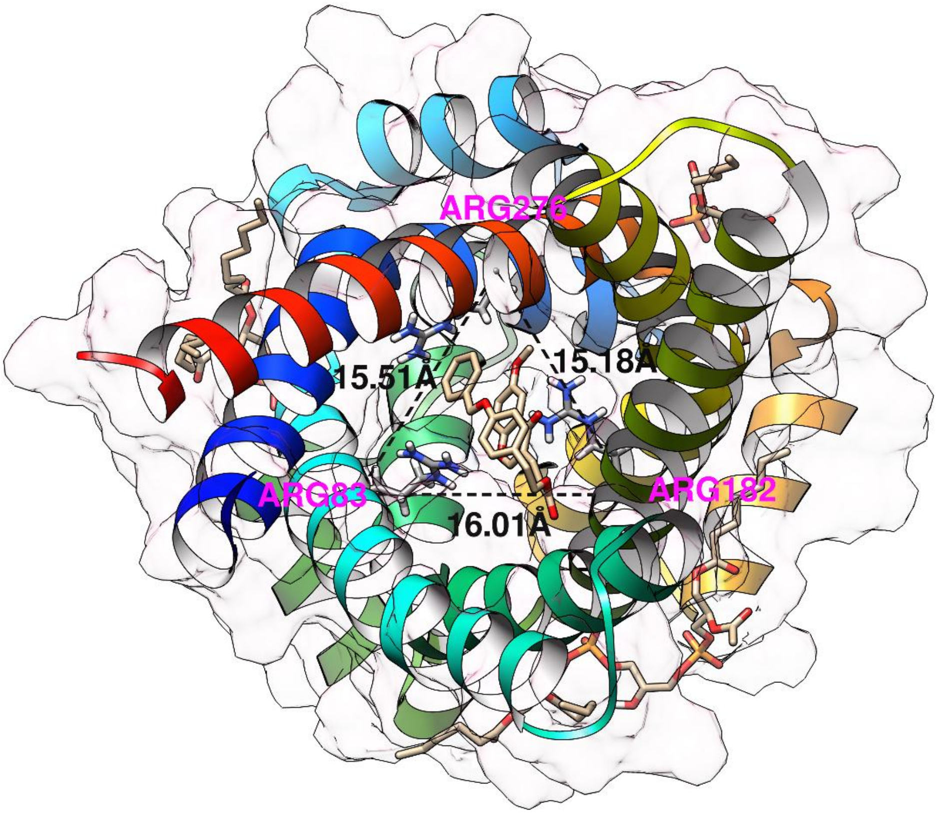

Figure S23. Structural positioning of BITSNPRG270 within the central cavity of UCP1

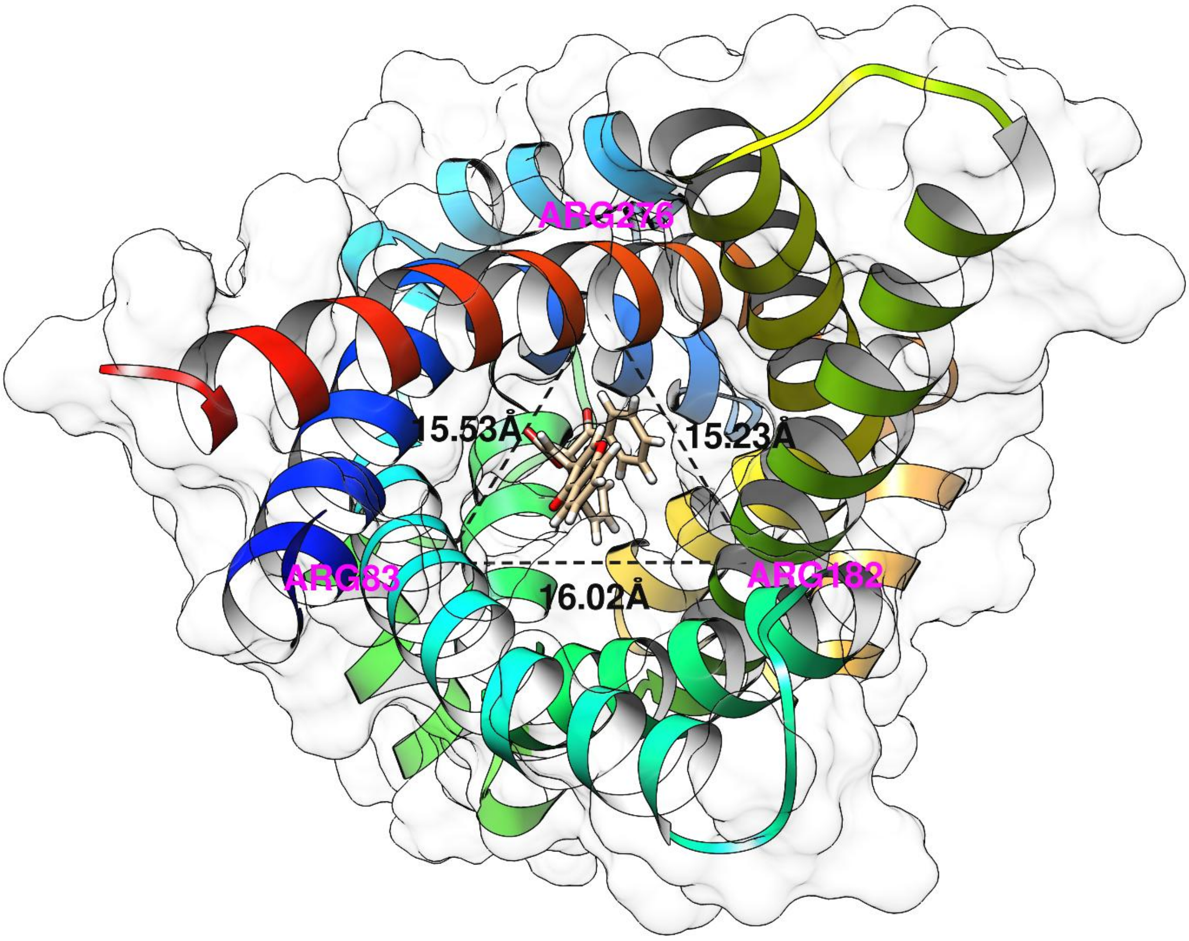

Figure S24. Structural positioning of BITSNPRG274 within the central cavity of UCP1

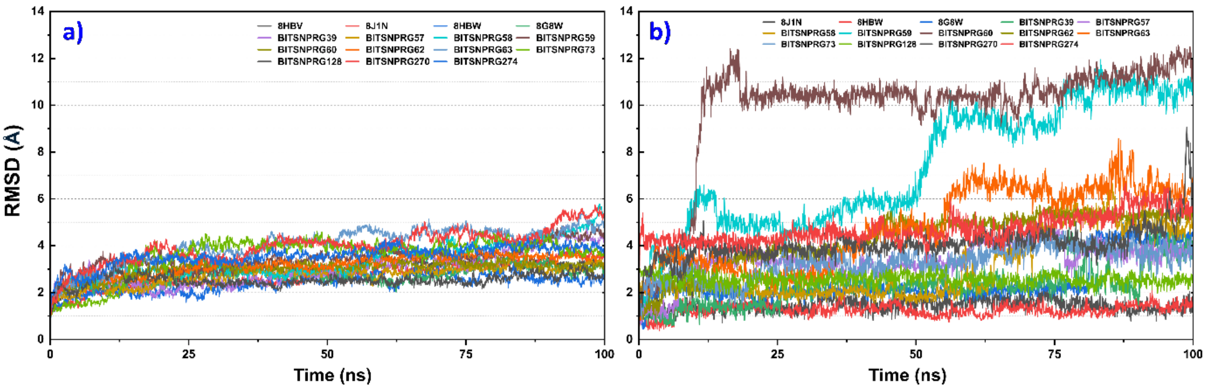

Figure S25. **Conformational pattern over 100 ns studied with RMSD**. **a**) Time evolution of RMSD profiles of UCP1 Cα atoms; **b**) Time evolution of RMSD profiles of native ligands (ATP, GTP, UTP, and DNP) and selected analogues bound in the UCP1 pocket.

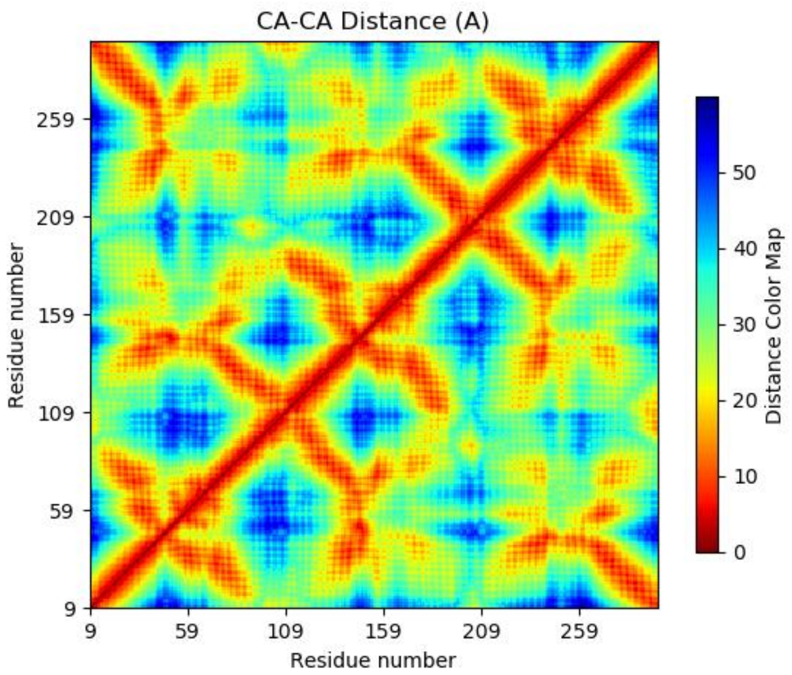

Figure S26. DCCMs for the UCP1 Cα atoms in complex with BITSNPRG270. The axes represent residue indices, and each matrix element indicates the degree of correlated motion between corresponding residue pairs. Both X- and Y-axes represent protein residue indices, depicting correlated motions between residue pairs. Warm colors (red to yellow) indicate positively correlated motions (moving in the same direction); Cool colors (green to blue) denote anti-correlated or negatively correlated motions (moving in opposite directions).

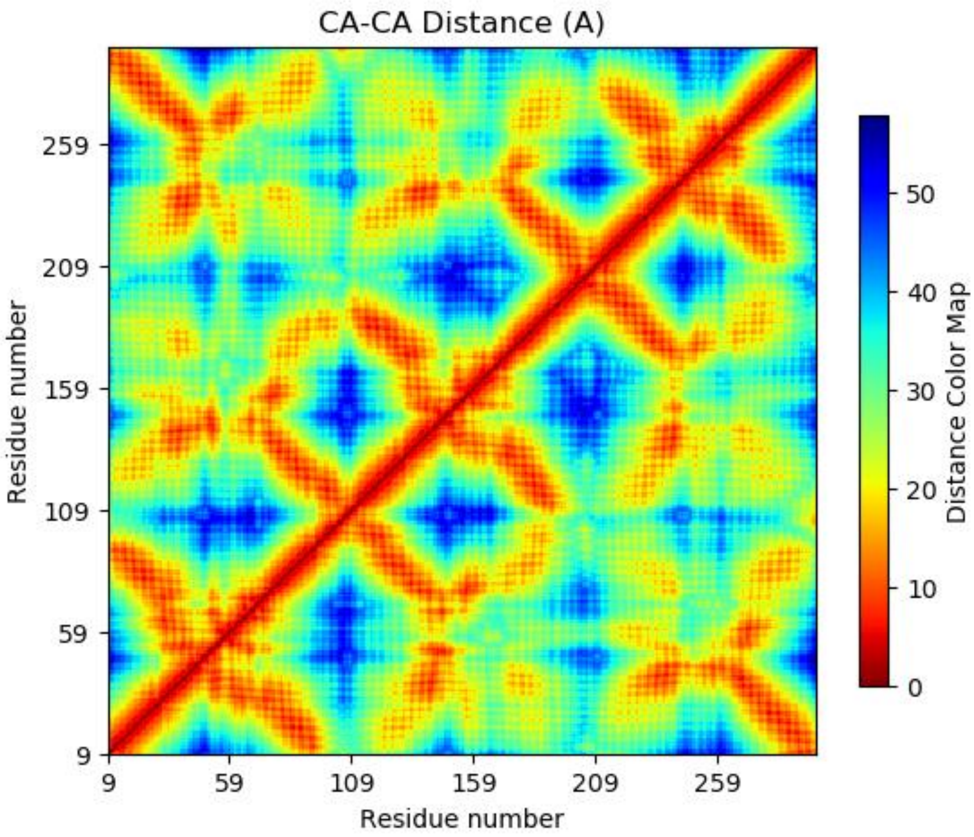

Figure S27. DCCMs for the UCP1 Cα atoms in complex with BITSNPRG274

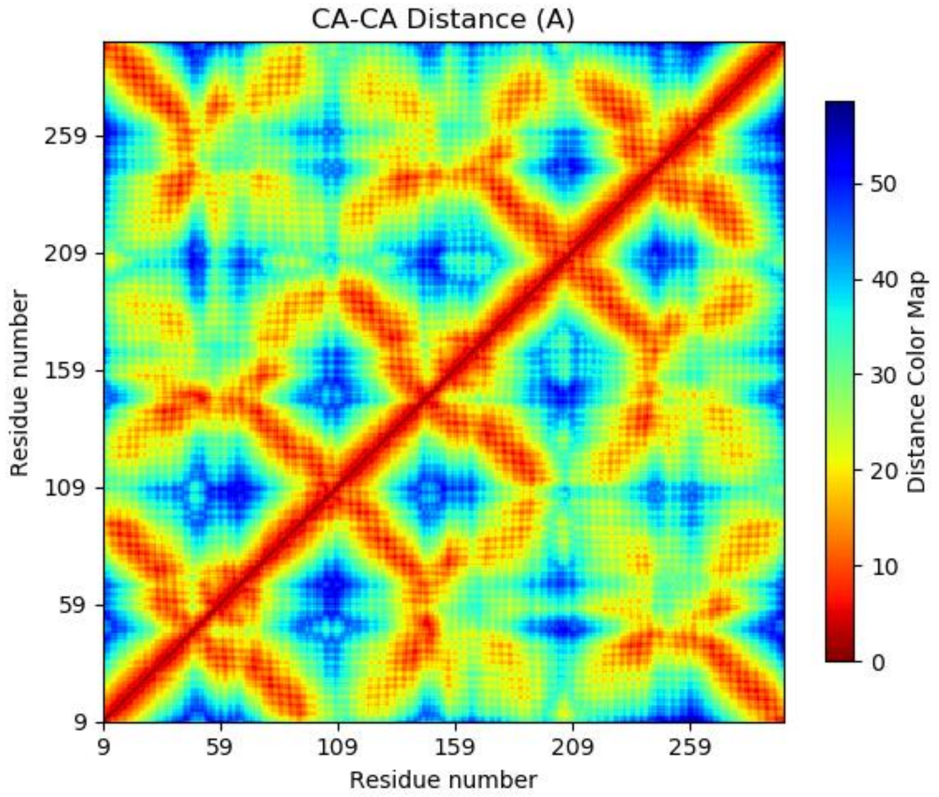

Figure S28. DCCMs for the UCP1 Cα atoms in complex with BITSNPRG128

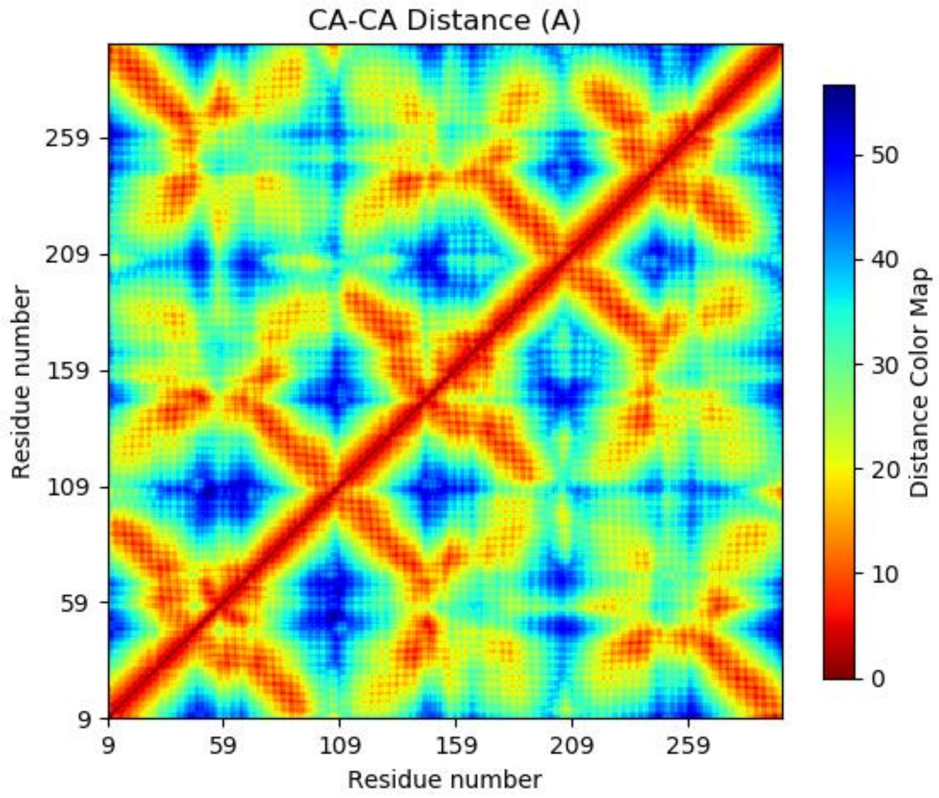

Figure S29. DCCMs for the UCP1 Cα atoms in complex with BITSNPRG39

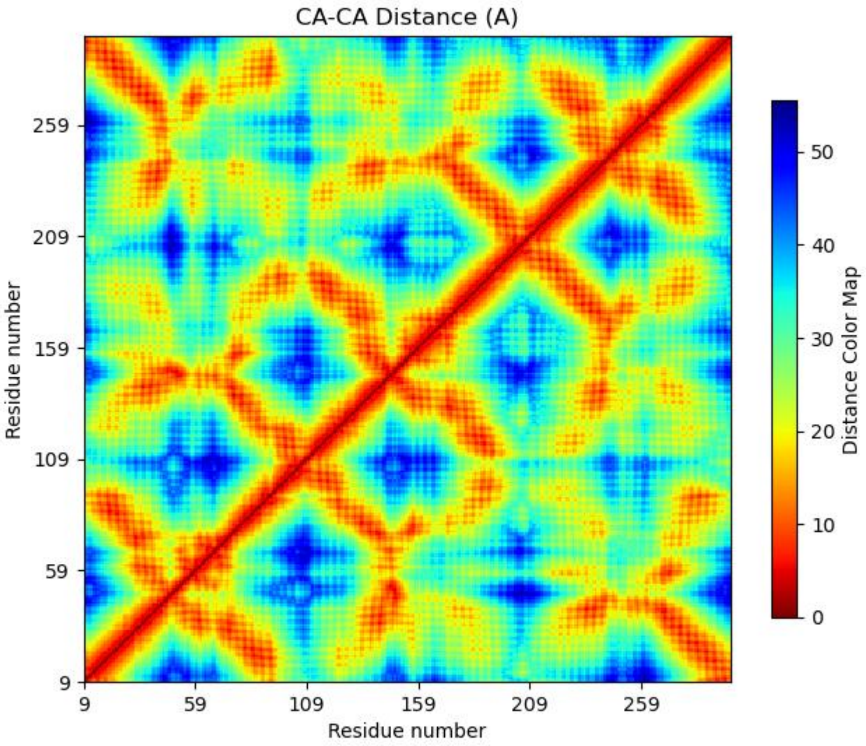

Figure S30. DCCMs for the UCP1 Cα atoms in complex with BITSNPRG57

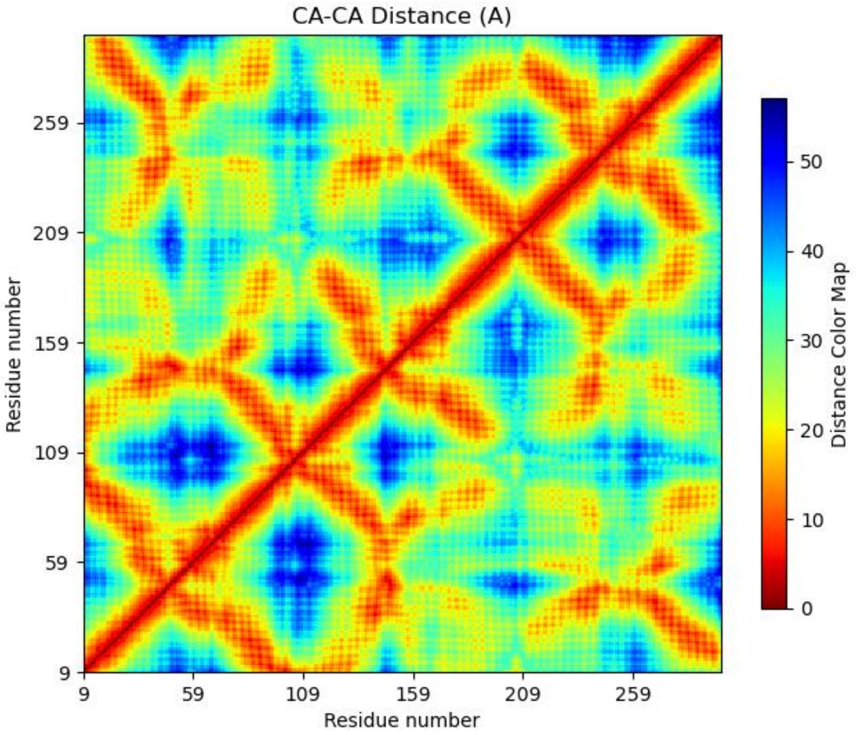

Figure S31. DCCMs for the UCP1 Cα atoms in complex with BITSNPRG58

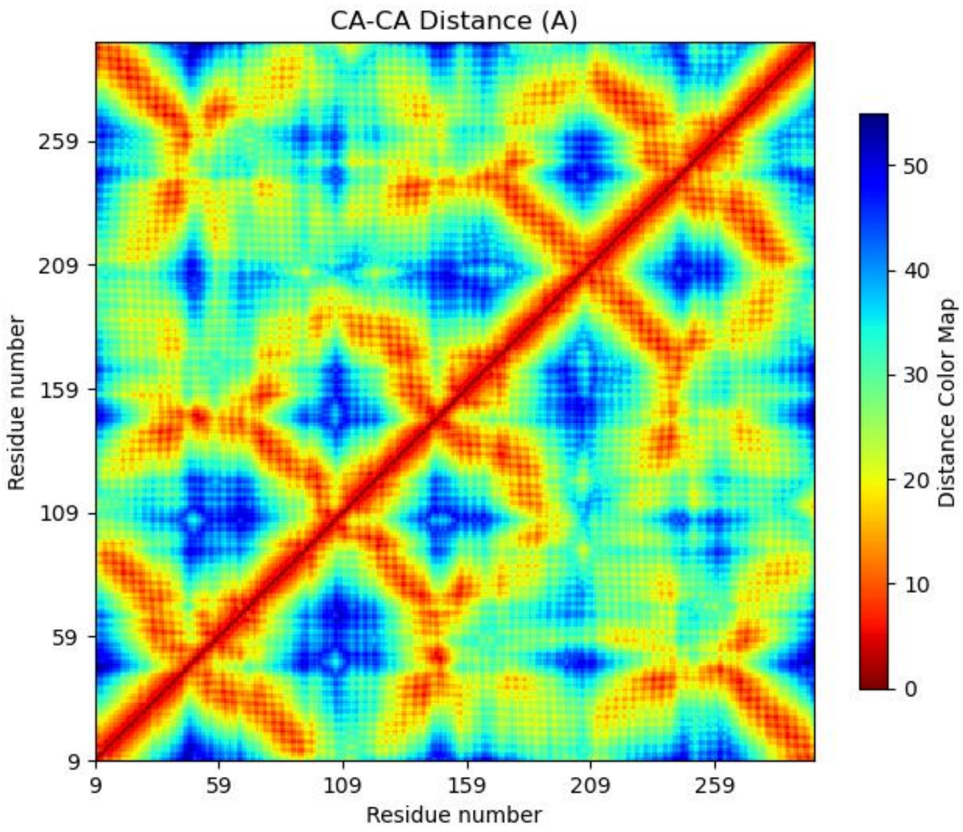

Figure S32. DCCMs for the UCP1 Cα atoms in complex with BITSNPRG59

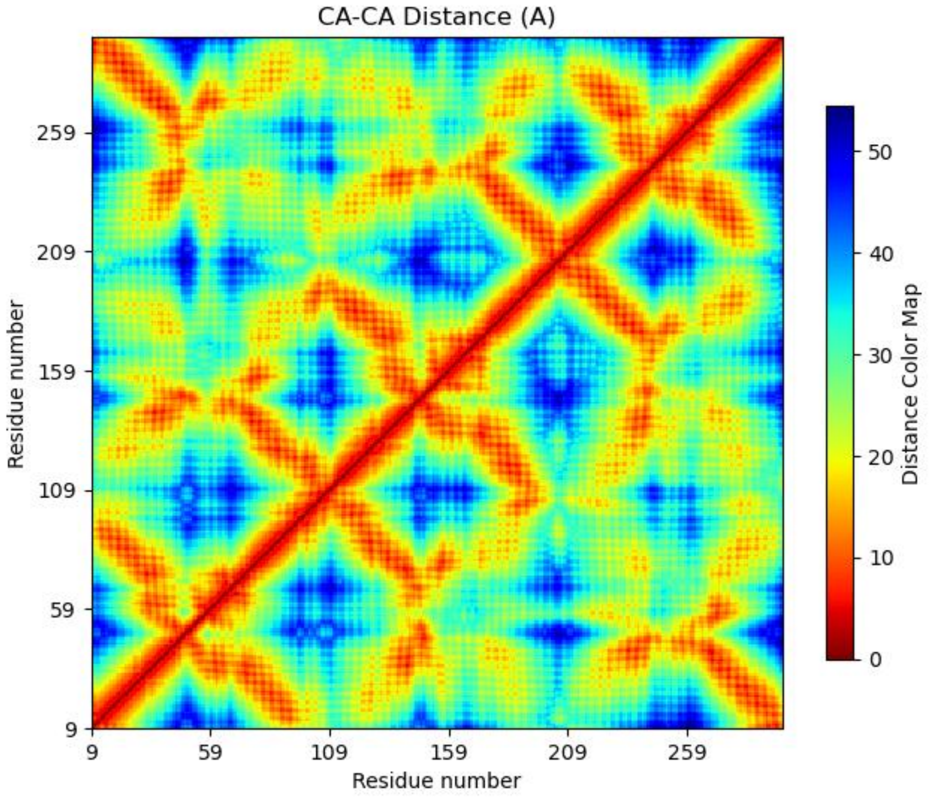

Figure S34. DCCMs for the UCP1 Cα atoms in complex with BITSNPRG60

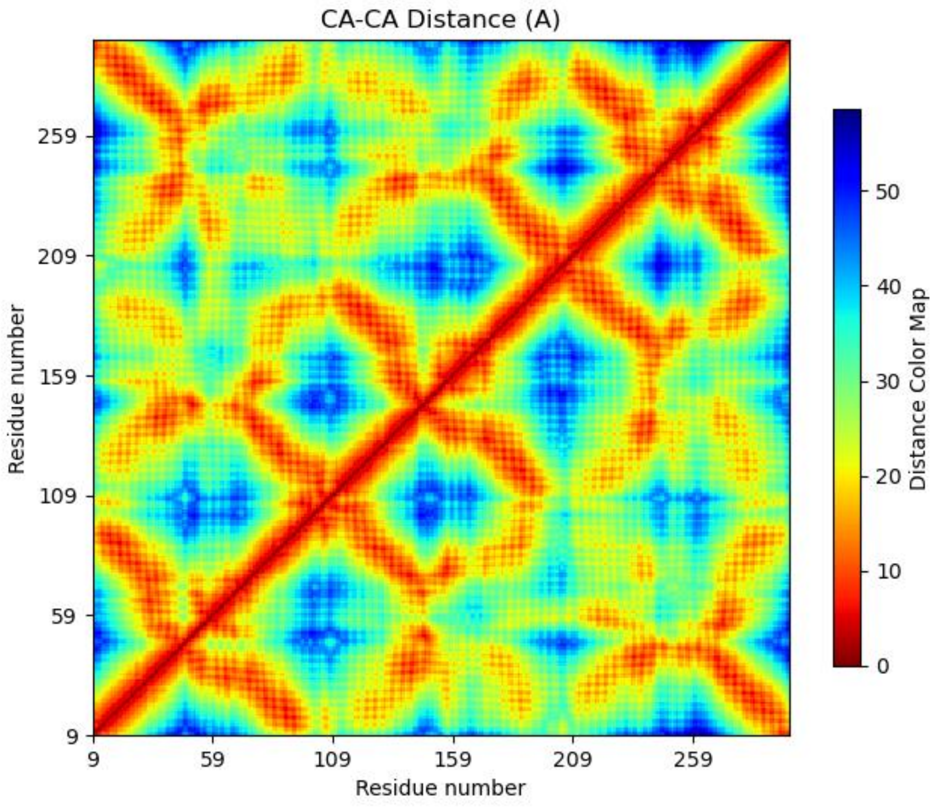

Figure S35. DCCMs for the UCP1 Cα atoms in complex with BITSNPRG62

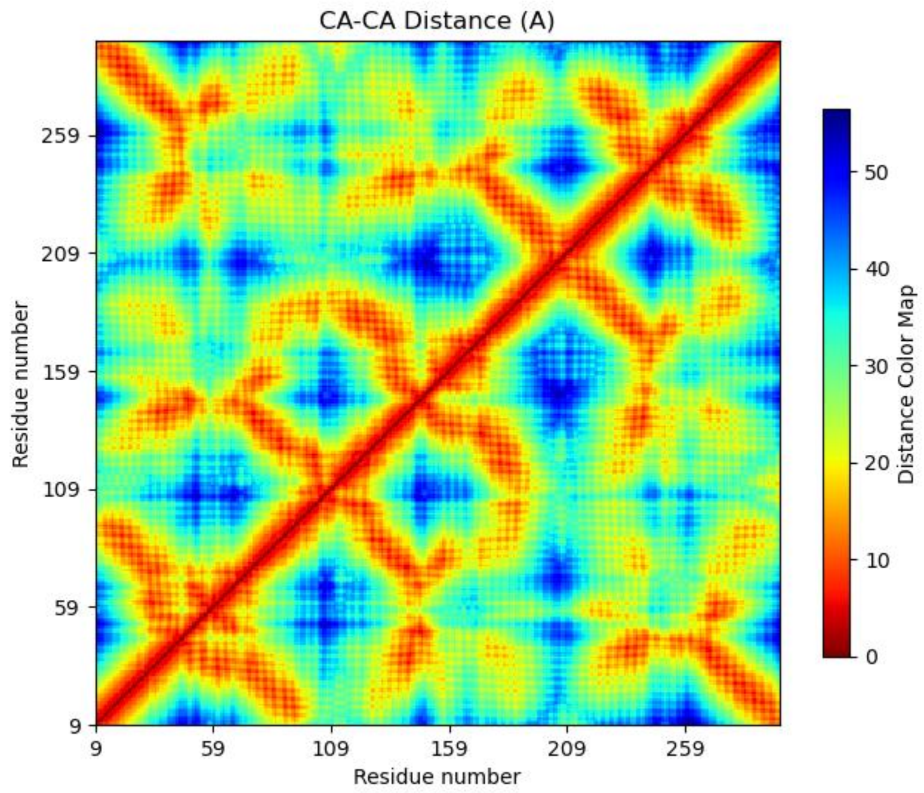

Figure S36. DCCMs for the UCP1 Cα atoms in complex with BITSNPRG63

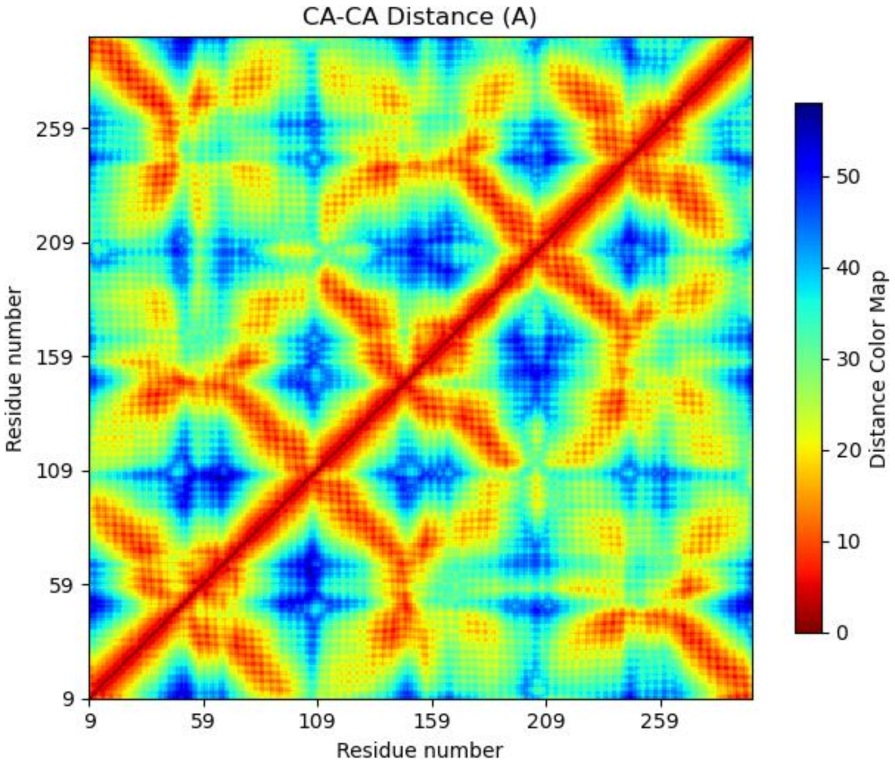

Figure S37. DCCMs for the UCP1 Cα atoms in complex with BITSNPRG73

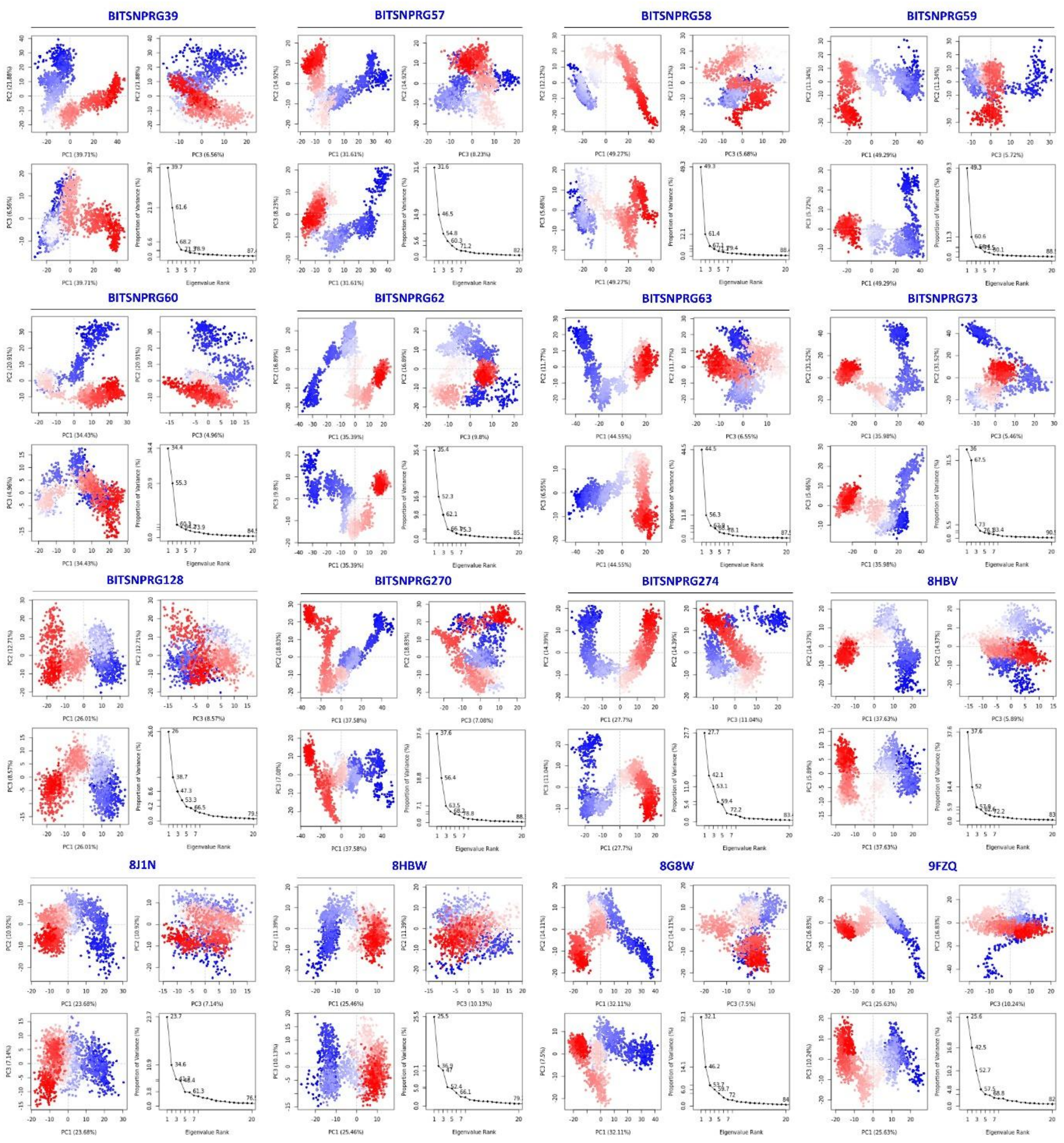

Figure S38. PCAs of UCP1 Cα atoms in the apo state and in complex with different ligands. For each complex, the scatter plots show the projections of the MD trajectories along the first three principal components (PC1 vs. PC2 and PC1 vs. PC3), highlighting the major conformational subspaces sampled during the simulation. Each dot represents a single conformer of the trajectory at a specific frame, color-coded from blue (early frames) to red (late frames), indicating the time evolution of structural changes. The rightmost plot in each group shows the scree plot of the eigenvalues, representing the variance explained by the top 20 principal components. The progressive color transition and clustering patterns reflect time-dependent conformational shifts.

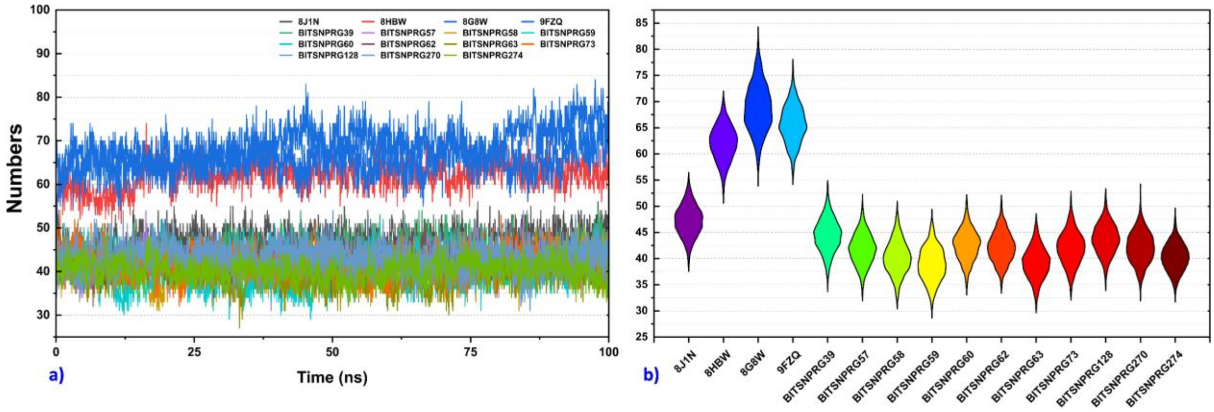

Figure 39. **Hydrogen bond profiling in simulated ligand-bound UCP1 complexes**. **a**) Number of hydrogen bonds over time for each complex over 100 ns simulations; b) Violin plots summarizing distributions of a number of hydrogen bonds across the trajectory for each complex.

Table S1. Calculated energies for FMOs and QCDs for the selected PL inhibitory analogues using the DFT method.

Entry	E_{HOMO} (eV)	E_{LUMO} (eV)	E_{HLgap} (eV)	DM (Debye)	IP (eV)	EA (eV)	χ (eV)	μ (eV)	η (eV)	ω (eV)
**BITSNPRG39**	-5.851	-1.446	4.405	5.861	5.851	1.446	3.649	-3.649	2.203	3.022
**BITSNPRG57**	-6.061	-1.626	4.435	7.073	6.061	1.626	3.844	-3.844	2.218	3.331
**BITSNPRG58**	-5.542	-1.444	4.098	4.103	5.542	1.444	3.493	-3.493	2.049	2.977
**BITSNPRG59**	-5.939	-1.487	4.452	2.942	5.939	1.487	3.713	-3.713	2.226	3.097
**BITSNPRG60**	-5.908	-1.525	4.383	6.196	5.908	1.525	3.717	-3.717	2.192	3.151
**BITSNPRG62**	-5.87	-1.399	4.471	2.866	5.87	1.399	3.635	-3.635	2.236	2.955
**BITSNPRG63**	-5.616	-1.555	4.061	3.983	5.616	1.555	3.586	-3.586	2.031	3.166
**BITSNPRG73**	-5.723	-1.565	4.158	5.359	5.723	1.565	3.644	-3.644	2.079	3.194
**BITSNPRG128**	-5.725	-1.752	3.973	7.661	5.725	1.752	3.739	-3.739	1.987	3.518
**BITSNPRG270**	-6.022	-2.09	3.932	4.265	6.022	2.09	4.056	-4.056	1.966	4.184
**BITSNPRG274**	-6.727	-2.258	4.469	3.469	6.727	2.258	4.493	-4.493	2.235	4.516

DM: dipole moment in Debye; IP: ionization potential in eV; EA: Electron affinity in eV; χ: electronegativity in eV; η: chemical hardness in eV; μ: chemical potential in eV; ω: electrophilicity in eV

Table S2. Critical parameters investigated in post-MD simulation analysis.

Category	Analysis	Purpose	Tool/module
**Conformational pattern, Flexibility, and Compactness**	RMSD	To assess structural stability and conformational changes over time	Simulation Interaction Diagram (SID), Schrödinger Desmond
	Radius of Gyration (RoG)	To evaluate changes in the overall size and compactness of the molecule	PyMol 3.0; Schrödinger Desmond
	Root Mean Square Fluctuation (RMSF)	To assess the flexibility and dynamics of specific regions or residues	SID, Schrödinger Desmond
**Arginine triplet dynamics**	Distance Mapping	Visualizes the distances and contacts between different parts of the system	PyMol 3.0; Schrödinger Desmond
**Dynamic Correlation and Motion Analysis**	Dynamic Cross-Correlation Map (DCCM)	To show the correlation of residual movements across the system	SID, Schrödinger Desmond

	Principal Component Analysis (PCA)	To capture and visualize major collective motions and dominant dynamic patterns	Bio3D in R integrated in Galaxy Australia Server
**Interaction Analysis**	Protein-ligand contacts	To map the interactions between the protein-ligand complex	SID, Schrödinger Desmond
	Interactions consistency	To evaluate the reliability and persistence of binding interactions	

[#]C-gate: Cystolic gate opening, M-gate: matrix gate opening

References

(1) Bursch, M.; Mewes, J. M.; Hansen, A.; Grimme, S. Best-Practice DFT Protocols for Basic Molecular Computational Chemistry**. *Angewandte Chemie International Edition* 2022, *61* (42), e202205735. https://doi.org/10.1002/ANIE.202205735.

(2) Wappett, D. A.; Goerigk, L. Benchmarking Density Functional Theory Methods for Metalloenzyme Reactions: The Introduction of the MME55 Set. *J Chem Theory Comput* 2023, *19* (22), 8365–8383. https://doi.org/10.1021/ACS.JCTC.3C00558.

(3) Anila Raj, S.; Vidya, V. G.; Preethi, V.; Viju Kumar, V. G. Single Crystal XRD and DFT Investigation of 1,5-Dimethyl-4-[(2-Oxo-1,2-Diphenylethylidene) Amino]-2-Phenyl-1,2-Dihydro-3H-Pyrazol-3-One. *Results Chem* 2022, *4*, 100665. https://doi.org/10.1016/J.RECHEM.2022.100665.
